# Defective TiO_2_ Nanotube Arrays for Efficient Photoelectrochemical Degradation of Organic Pollutants

**DOI:** 10.1021/acsomega.3c00820

**Published:** 2023-06-07

**Authors:** Manel Machreki, Takwa Chouki, Georgi Tyuliev, Dušan Žigon, Bunsho Ohtani, Alexandre Loukanov, Plamen Stefanov, Saim Emin

**Affiliations:** † Materials Research Laboratory, University of Nova Gorica, Vipavska 11c, 5270 Ajdovščina, Slovenia; ‡ Institute of Catalysis, Bulgarian Academy of Sciences, Acad. G. Bonchev St., Bldg. 11, Sofia 1113, Bulgaria; § Institute “Jožef Stefan”, Jamova 39, 1000 Ljubljana, Slovenia; ∥ Catalysis Research Center, Hokkaido University, N21, W10, 001-0021 Sapporo, Japan; ⊥ Department of Chemistry and Materials Science, National Institute of Technology, Gunma College, 580 Toriba, Maebashi 371-8530, Gunma, Japan; # Institute of General and Inorganic Chemistry, Bulgarian Academy of Sciences, Sofia 1113, Bulgaria

## Abstract

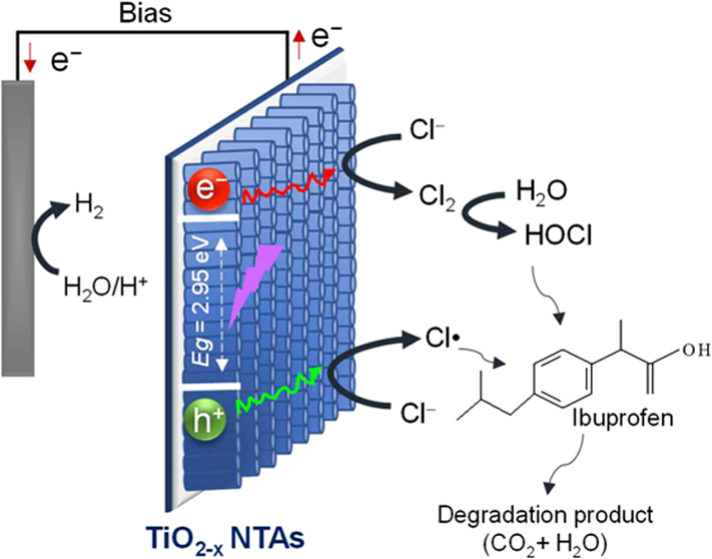

Oxygen vacancies (OVs) are one of the most critical factors that enhance the electrical and catalytic characteristics of metal oxide-based photoelectrodes. In this work, a simple procedure was applied to prepare reduced TiO_2_ nanotube arrays (NTAs) (TiO_2–*x*_) via a one-step reduction method using NaBH_4_. A series of characterization techniques were used to study the structural, optical, and electronic properties of TiO_2–*x*_ NTAs. X-ray photoelectron spectroscopy confirmed the presence of defects in TiO_2–*x*_ NTAs. Photoacoustic measurements were used to estimate the electron-trap density in the NTAs. Photoelectrochemical studies show that the photocurrent density of TiO_2–*x*_ NTAs was nearly 3 times higher than that of pristine TiO_2_. It was found that increasing OVs in TiO_2_ affects the surface recombination centers, enhances electrical conductivity, and improves charge transport. For the first time, a TiO_2–*x*_ photoanode was used in the photoelectrochemical (PEC) degradation of a textile dye (basic blue 41, B41) and ibuprofen (IBF) pharmaceutical using in situ generated reactive chlorine species (RCS). Liquid chromatography coupled with mass spectrometry was used to study the mechanisms for the degradation of B41 and IBF. Phytotoxicity tests of B41 and IBF solutions were performed using *Lepidium sativum* L. to evaluate the potential acute toxicity before and after the PEC treatment. The present work provides efficient PEC degradation of the B41 dye and IBF in the presence of RCS without generating harmful products.

## 1 Introduction

The photoelectrochemical (PEC) approach has emerged as a promising oxidation process for the degradation of organic pollutants in wastewater and indoor air.^1,2^ In this technique, a bias potential is applied to promote the separation of the photogenerated electron and hole pairs in each semiconductor film. Titanium dioxide (TiO_2_) has been recognized as a suitable material for PEC wastewater treatment because it is nontoxic, low-cost, and chemically stable against corrosion.^3−7^ TiO_2_ in the form of nanotube arrays (NTAs) has been demonstrated to offer advantages in PEC oxidation processes due to the following reasons: (i) it exhibits a large surface area which provides more reaction sites, (ii) sufficient pore sizes, (iii) reduced light reflection, and (iv) improved charge recombination.^8^ However, the large band gap (∼3.2 eV) of TiO_2_ and the high recombination rate of photoinduced charge carriers make its PEC efficiency unsatisfactory.^9^ Therefore, different methods have been explored to reduce both the band gap and/or the recombination of photoinduced charge carriers in TiO_2_, including the preparation of heterostructures, heteroatom doping, deposition of noble metals, etc.^3^ Oxygen vacancy (OV) engineering has been found as another approach to narrow the band gap of TiO_2_ and improve its PEC performance.^10^

In recent years, TiO_2–*x*_ NTAs that contain OVs (e.g., Ti^3+^) have attracted increasing attention due to their improved transport and photoelectric properties in batteries,^11^ field-emission devices,^12^ supercapacitors,^13^ water-splitting studies,^14^ and degradation of organic pollutants.^15^ For example, because of OVs, the field-emission properties of TiO_2_ can be enhanced, and the turn-on field could reach down to 1.75 V μm^–1^.^16^ The OVs in TiO_2_ are known to be shallow donors with relatively low formation energies.^11,13,17^ Different strategies have been developed to introduce OVs in TiO_2_ lattices, such as hydrogenation, thermal reduction, electrochemical reduction, or liquid-phase reduction.^18^ However, some of these strategies require high temperatures, expensive equipment, or harsh synthetic conditions, which limit their widespread use. Xing et al. have found that TiO_2–*x*_ nanoparticles can be facilely prepared using a reducing reagent such as sodium borohydride (NaBH_4_) in a liquid-phase environment.^19^ The authors found that the reduction of some Ti^4+^ atoms to Ti^3+^ is facilitated by atomic hydrogen, which creates oxygen vacancies in the TiO_2_ lattice.^14,20^ The advantage of the NaBH_4_ reduction method over the conventional hydrogenation approach at elevated temperatures is that it eliminates the risk of a gas explosion.

Ibuprofen (IBF) is a widely used analgesic anti-inflammatory drug. It has been detected in municipal wastewater treatment plant effluents and natural waters due to its wide use and biodegradation resistance.^21−23^ IBF accumulation in wastewaters has been shown to have an irreversible negative effect on frog embryos or catfish plasma.^24^ When combined with other non-steroidal anti-inflammatory medicines, IBF may have ecotoxicological impacts.^25^ Similarly, the basic blue 41 (B41) dye was chosen because it is a toxic effluent in the textile industry.^26^ Therefore, it is important to eliminate both B41 and IBF molecules from wastewater before discharging them into the ecosystem. The removal of these contaminants from synthetic water streams was explored using approaches like UV photolysis,^27^ photocatalysis,^23,28−30^ and electrocatalysis.^31^ So far, few studies deal with PEC removal of contaminants like B41 and IBF from wastewaters using metal oxides.^21,32^ Furthermore, there is a lack of information about the use of metal oxides like TiO_2_ NTA with engineered OVs in the PEC treatment of organics from contaminated waters.

In the present study, we applied the NaBH_4_ reduction method to produce TiO_2–*x*_ NTAs at room temperature, which allowed the generation of OVs. The defects allow for improved charge transport, increased charge injection, and faster surface catalytic kinetics under the PEC system. Moreover, the pristine TiO_2_ and TiO_2–*x*_ NTAs were investigated using different techniques like reverse double-beam photoacoustic spectroscopy (RDB-PAS) (to estimate the electron-trap density), photoelectrochemical techniques, X-ray diffraction, transmission electron microscopy, scanning electron microscopy, and X-ray photoelectron spectroscopy. To the best of our knowledge, for the first time, we applied the TiO_2–*x*_ NTAs as photoanodes for PEC degradation of the B41 dye and IBF pharmaceutical in the presence of in situ generated reactive chlorine species (RCS) such as chlorine radicals (Cl^•^, Cl_2_^•–^), chlorine (Cl_2_), hypochlorite ion (ClO^–^), and hypochlorous acid (HOCl). Liquid chromatography-mass spectrometry (LC-MS) was used to monitor the degradation of these organics and their by-products. Chemical oxygen demand analyses were carried out to evaluate the mineralization levels. Various operation parameters, including anodic potential, solution pH, electrolytes, and concentration of pollutants, were investigated to determine the optimal conditions for the degradation of pollutants. A phytotoxicity test was carried out using *Lepidium sativum* L. to evaluate the potential acute toxicity of the original and treated B41 and IBF pollutants.

## 2 Experimental Section

### 2.1 Chemicals

Titanium foil (0.2 mm thick, 99.9% purity) was obtained from Ankuro Int. GmbH (Germany). Nitric acid (HNO_3_, 65%) was bought from Carlo Erba Reagents GmbH (Germany). Hydrofluoric acid (HF, 48–51%) and furfuryl alcohol (FFA, 99%) were purchased from Fisher Scientific. Ethylene glycol (99%), ammonium fluoride (NH_4_F, 99.8%), sodium borohydride (NaBH_4_, 97%), sodium chloride (NaCl, 99%), sodium sulfate (Na_2_SO_4_, >99%), Ibuprofen (IBF, ≥98%), coumarin, and sodium hypochlorite (NaOCl) were purchased from Alfa Aesar (U.K.). Ammonium chloride (NH_4_Cl, 98%) and 7-hydroxycoumarin were obtained from Acros Organics. Ethylenediaminetetraacetic acid (EDTA), sodium thiosulfate (Na_2_S_2_O_3_), and *tert*-butyl alcohol (TBA) were purchased from Sigma-Aldrich. A graphite block (99.9%) was purchased from Beijing Great Wall Co., Ltd. (China). Deionized water was used in all of the experiments.

### 2.2 Preparation of TiO_2_ Nanotubes

The TiO_2_ nanotubes were prepared by the anodization method. First, we polished a titanium foil with different abrasive papers.^33^ Followed by cleaning in an ultrasound bath using acetone and ethanol for 5 min and deionized water for 10 min in turn. The titanium (Ti) foil was chemically etched in a mixture solution of hydrofluoric acid (HF) and nitric acid (HNO_3_) (HF/HNO_3_/H_2_O = 1:4:5 in volume, a total of 20 mL) for 30 s and washed with water. The anodization process was performed in a two-electrode system using Pt as the counter electrode (CE) and Ti foil as the working electrode (WE) at 30 V for 2 h. The electrolytes were 2.5% H_2_O (2.5 mL) and 0.28 wt % NH_4_F in ethylene glycol (97.5 mL). After anodization, the film was sonicated in methanol for a short time, rinsed with deionized water, and finally dried with a nitrogen (N_2_) stream. The obtained TiO_2_ film was annealed at 500 °C for 1 h in air. The heating rate was 2 °C min^–1^. The annealed samples were dipped in 1 M NaBH_4_ solution for different times (0–16 h) with the goal of producing TiO_2–*x*_ NTA.

### 2.3 Material Characterization

The X-ray diffraction (XRD) pattern of the TiO_2_ NTA films was recorded within the 2θ range from 10 to 90° with a constant step of 0.03° using a MiniFlex 600 W (Rigaku) diffractometer (Cu Kα radiation). Phase identification was studied with PDXL software using the crystallography open database. The absorption studies were performed using a Lambda 650 UV–VIS spectrophotometer (PerkinElmer). The morphology of TiO_2_ films was studied using a scanning electron microscope JSM 7100F SEM (JEOL) equipped with a field-emission electron gun. Energy-dispersive X-ray spectroscopy (EDS) analysis was performed at 10 kV using an EDS detector (Oxford Instruments) attached to the SEM machine. Transmission electron microscopy (TEM) studies were studied with a JEOL 2100F. EC and PEC measurements were carried out using a Cappuccino cell using a three-electrode system. An O-ring was used to define the electrode area to 0.283 cm^2^. The working electrode (WE) potential was controlled by a potentiostat (EDAQ SP1). A graphite plate was used as the counter electrode (CE), and Hg/Hg_2_SO_4_ as a reference electrode (RE). The electrochemical impedance spectroscopy (EIS) of TiO_2_ nanotubes was studied under light and dark in 0.1 M Na_2_SO_4_ solution. The applied frequency ranged from 1 Hz to 100 kHz with an amplitude of 5 mV. The measured EIS data were fitted using the Zview software. The Mott–Schottky (M–S) plots were derived from impedance potential tests conducted at a frequency of 1 kHz in the dark. Details of the RDB-PAS measurements and XPS studies are provided in the Supporting Information.^34^

### 2.4 PEC Degradation of B41 and IBF

The degradation of B41 and IBF molecules (Table S1) was performed in a two-compartment PEC cell comprising anodic (75 mL) and cathodic (55 mL) chambers separated by a proton exchange membrane (Nafion-115) (Figure S1). All of the experiments were conducted using TiO_2–*x*_ NTAs as the WE (area 6 cm^2^), the Hg/Hg_2_SO_4_ electrode as the RE, and a graphite plate as the CE. A distance of 3 cm was kept between the WE and the CE. The degradation of organic substances (14.4 mg L^–1^ B41 and 45.6 mg L^–1^ IBF) were carried out using different electrolytes (such as NaCl, NH_4_Cl, and Na_2_SO_4_). The light irradiation source (λ_ex_: 370 nm, 20 W) was utilized to illuminate the PEC cell. The incident light intensity (2.6 mW cm^–2^) was determined using an optical power meter (Thorlabs PM320E Dual-Channel Optical Power & Energy Meter Console). The spectrum of the light source is given in Figure S1e. B41 dye and IBF degradation at different reaction times were studied using a PerkinElmer Lambda 25 UV–VIS spectrophotometer. The chemical oxygen demand (COD) of B41 dye solutions was determined using a colorimetric approach with K_2_Cr_2_O_7_ (Macherey Nagel, Nanocolor CSB 160). The hydroxyl radical (^•^OH) generation during the PEC system was done using coumarin as a trapping reagent with a photoluminescence spectrometer (FL920 spectrophotometer, Edinburgh Instruments). The degradation of intermediate products of IBF and the B41 dye during the PEC process was checked by LC-MS measurements using mass spectrometer Q-Tof Premier interfaced to the ultra-performance liquid chromatograph (UPLC) system based on Waters Acquity (Waters, Milford) (LC-MS conditions described in the Supporting Information).

## 3 Results and Discussion

### 3.1 Characterization of the Samples

**Figure 1:**
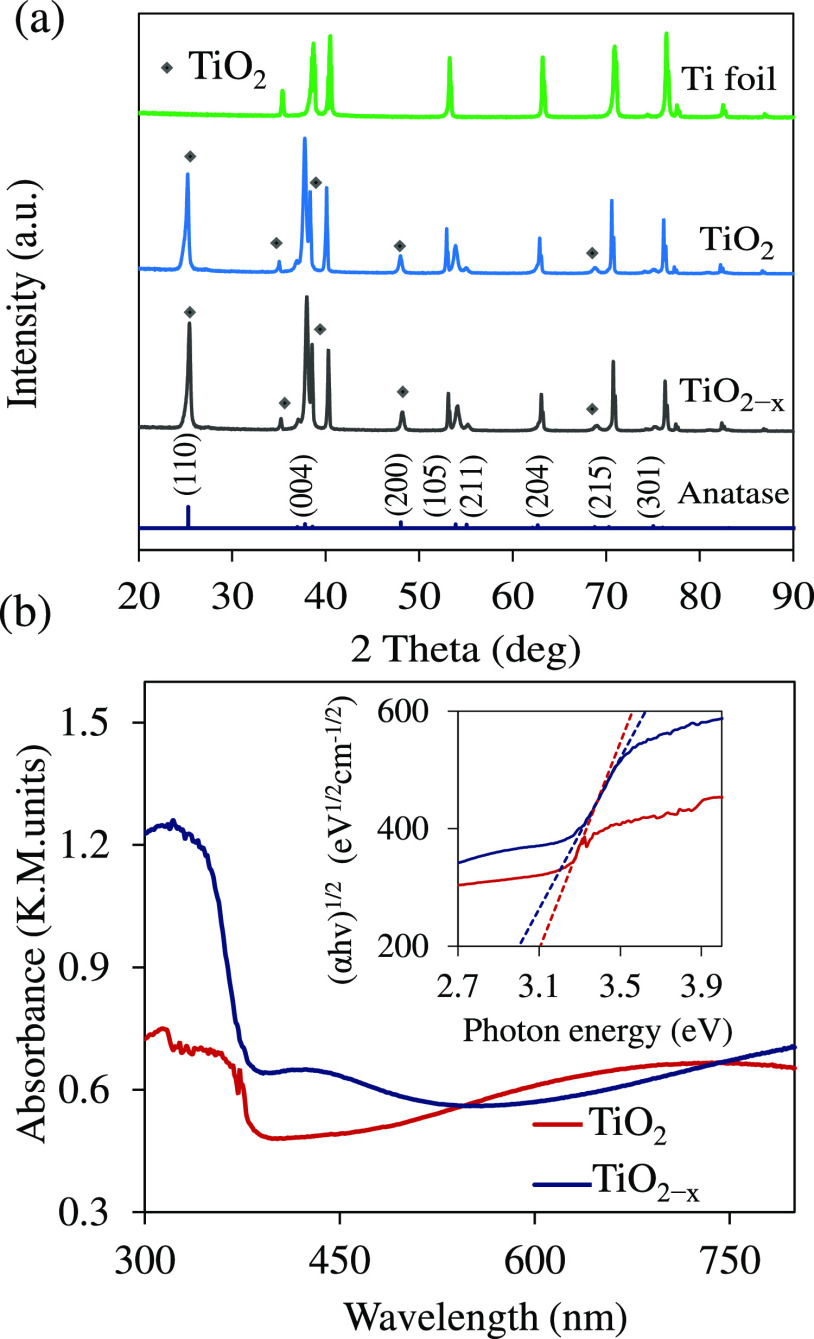
(a) XRD patterns and (b) absorbance spectra of TiO_2_ and TiO_2–*x*_ NTAs (in 1 M NaBH_4_ solution for 16 h). The inset in (b) is a Tauc plot.

XRD patterns of the TiO_2_ and the TiO_2–*x*_ NTA films are shown in Figure 1a. All of the diffraction peaks of the TiO_2–*x*_ NTAs match the anatase phase of TiO_2_ (I41 (141); PDF# 9008213).

An insignificant change in the diffraction peaks of the pristine TiO_2_ NTAs was observed after chemical reduction. The diffraction peaks at 25.28, 37.80, 48.05, 53.89, 62.30, and 75.03° correspond to the (101), (004), (200), (105), (204), and (215) planes of the anatase TiO_2_ phase.^14^ The remaining peaks in the diffractogram belong to the titanium foil.

UV–VIS spectroscopy was used to determine the optical band gap (*E*_g_) of NTA films (Figure 1b). The TiO_2–*x*_ NTAs show a pronounced absorption below 500 nm. Using the Tauc equation and assuming an indirect transition, the *E*_g_ is estimated from the following relation: (α*hv*)^1/*n*^ = *A*(*hv* – *E*_g_), where α is the linear absorption coefficient of the material, *hv* is photon energy, and *A* is a proportionality constant.^32^ The *E*_g_ values of the pristine TiO_2_ and TiO_2–*x*_ NTA films taken from the intercept are equal to ∼3.1 and 2.95 eV, respectively (Figure 1b inset). This decrease in the band gap in the TiO_2–*x*_ NTA sample is consistent with literature reports.^17^

**Figure 2:**
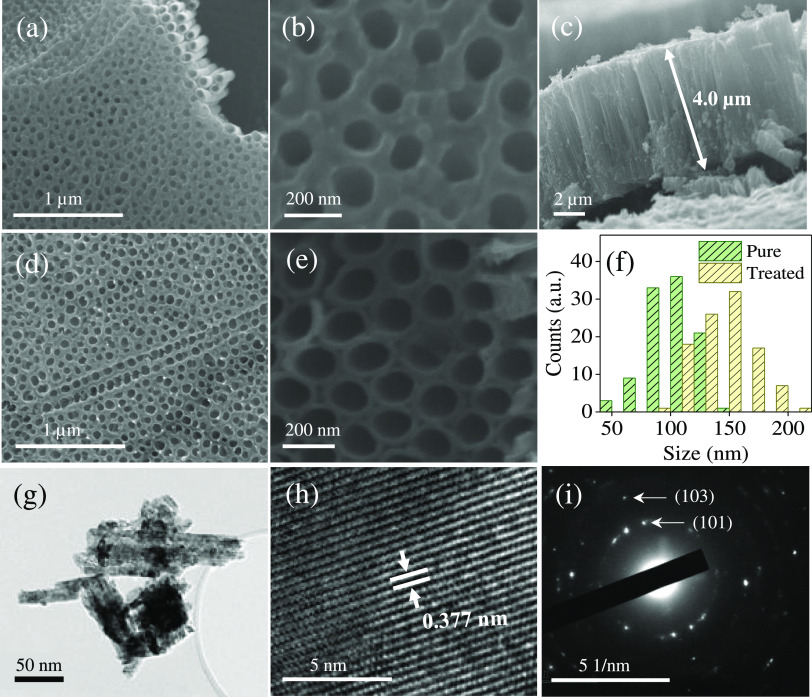
(a, b) SEM images show the TiO_2_ NTAs at different magnifications. (c–e) Cross-section and top-view images of the TiO_2–*x*_ NTAs obtained in 1 M NaBH_4_ solution for 16 h. (f) Size distribution of TiO_2_ and TiO_2–*x*_ NTAs treated. (g) The TEM image of TiO_2_ tubes. (h) The HR-TEM image of the TiO_2_ lattice and (i) the SAED pattern taken from (g).

Figure 2a–f shows typical SEM images of pristine TiO_2_ and TiO_2–*x*_ NTAs. It is evident that the pristine TiO_2_ NTAs exhibit porous and uniform morphology on top and top-open nanotubular structure underneath, as revealed in Figure 2a,b. The inner diameter of the tubes and film thickness of the TiO_2–*x*_ NTAs are estimated to be in the order of ∼100 nm and ∼4.0 μm, respectively (Figure 2c). Reduction of TiO_2_ NTAs in 1 M NaBH_4_ solution for 16 h preserved the pore in TiO_2_ NTAs. However, closer observation reveals that the TiO_2–*x*_ nanotubes separate from each other, and the wall gets thinner, as shown in Figure 2f. This indicates that NaBH_4_ reacted with TiO_2_. In the aqueous medium, hydrolysis of NaBH_4_ produces reducing hydrogen, which in turn reduces Ti^4+^ to Ti^3+^^19^
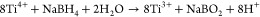
1

The average diameter of TiO_2–*x*_ NTAs is higher than that obtained for pure TiO_2_, which falls within the range of 50–100 nm. According to a previous report, thinner walls benefit the charge transport in TiO_2_ NTAs.^14^ We can infer that the TiO_2–*x*_ NTAs may offer a higher photocatalytic activity. A scratch made on the TiO_2_ NTAs allowed us to collect materials on the TEM grid. Figure 2g shows an agglomerate composed of TiO_2_ tubes. High-resolution TEM indicated that the lattice spacing is equal to 0.377 nm, which corresponds to the (101) crystal plane of anatase TiO_2_ (Figure 2h). The pattern of selected area electron diffraction (SAED) shows that these particles are polycrystalline (Figure 2i).^35^ The diffraction rings in the SAED can be indexed to the (101) and (103) of TiO_2_ NTAs.

**Figure 3:**
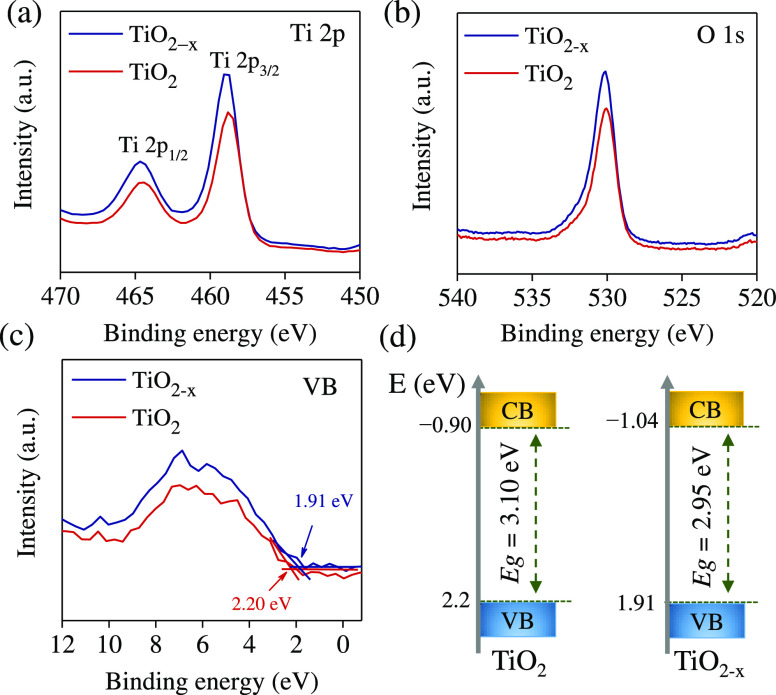
XPS spectra of pristine TiO_2_ and TiO_2–*x*_ treated in NaBH_4_ solution for 16 h. (a) Ti 2p, (b) O 1s, and (c) XPS valence band spectra. (d) Schematic illustration of the estimated position of the VB and the CB for pristine TiO_2_ and TiO_2–*x*_.

XPS was employed to investigate the effect of reduction treatment on the chemical composition and oxidation state in pristine and reduced TiO_2_ samples. The survey XPS spectra are given in Figure S2a. The narrow XPS scan of Ti 2p and O 1s are shown in Figure 3a,b. For the pure TiO_2_, the peaks at 464.43 and 458.8 eV can be attributed to Ti 2p_3/2_ and Ti 2p_1/2_, respectively. These feature peaks belong to the Ti^4+^–O bonds in TiO_2_ NTAs.^36^ In the case of the TiO_2–*x*_ NTA sample, the Ti 2p peaks are slightly shifted to 458.9 and 464.67 eV, respectively (Figure 3a). These shifts may be attributed to the presence of Ti^3+^ or OVs in the nanocrystals.^14,37,38^ The shape of the O 1s XPS signal in these samples shows small differences (Figure 3b). XPS spectra for O 1s data were fitted with three Gaussian curves, which are attributed to lattice oxygen (O_L_), oxygen vacancies (O_V_), and chemisorbed oxygen species (O_C_), respectively (Figure S2b,c).^14^ The low binding energy peak (O_L_) comes from the lattice oxygen atoms (O^–2^) in a fully coordinated TiO_2_ with the Ti^4+^ ions, mainly in the bulk. The medium binding energy peak (O_V_) is attributed to hydroxyl species due to the adsorption of moisture at OVs at the TiO_2_ surface, and the high binding energy peak (O_C_) is the loosely adsorbed, dissociated oxygen or O_2_ and H_2_O at the surface of TiO_2_.^37^ Furthermore, the concentrations of OVs in the TiO_2_ films with and without the reduction process were estimated. As shown in Figure S2d, The relative concentrations of the OVs were gradually increase with the chemical reduction time.^36^ While the O_C_ peaks are exponentially improved throughout the reduction time, this implies that the loose chemisorbed oxygen species on the surface increase. Energy-dispersive X-ray spectroscopy (EDS) analysis was also used as a rough estimate to determine the atomic ratios of O and Ti elements (Figure S2e,f). The density of states (DOS) of the valence band (VB) of TiO_2_ and TiO_2–*x*_ TNAs were determined from the valence band XPS data (Figure 3c). Pure TiO_2_ TNAs show typical TiO_2_ valence band DOS characteristics with the highest energy edge at 2.20 eV under the Fermi energy. Since the optical band gap of pure TiO_2_ TNAs is 3.1 eV (Figure 1b inset), the conduction band (CB) minimum would occur at −0.9 eV. On the other hand, the VB maximum energy of reduced TiO_2–*x*_ TNAs has shifted slightly to 1.91 eV. The CB minimum of the reduced TiO_2–*x*_ TNAs would occur at −1.04 eV, based on the results of optical measurements, which indicate a narrower band gap. Figure 3d shows a schematic representation of the energy diagram of pure and reduced TiO_2_ TNAs. Furthermore, the presence of oxygen vacancies in the crystal structure of TiO_2–*x*_ NTA is thought to be the cause of this change.^18^

**Figure 4:**
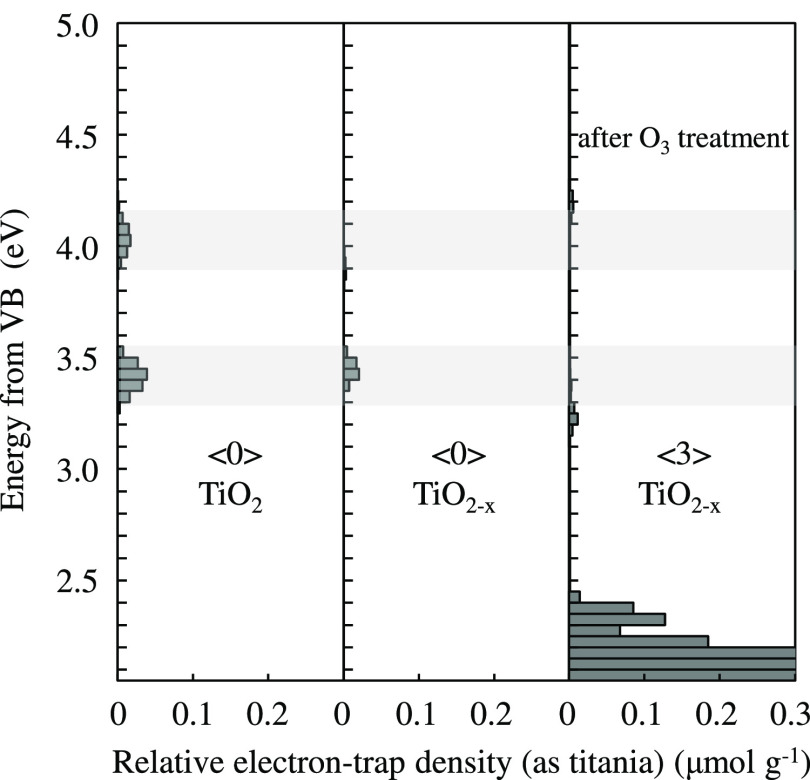
Representative ERDT patterns of TiO_2_ and TiO_2–*x*_ NTAs. Figures in < > denote the total density of electron-trap density in units of μmol g^–1^.

Photoacoustic measurements were found to be useful in understanding the behavior of photoinduced electrons and holes in our samples. RDB-PAS studies were conducted to further understand defect distribution in TiO_2_ and TiO_2–*x*_ NTAs. RDB-PAS can measure the energy-resolved distribution of electron traps (ERDT) and provide valuable information on the density of electron transitions from the top of the VB to existing electron traps.^39^ The electron-trap density in the pristine TiO_2_ NTAs was found to be higher than in the case of TiO_2–*x*_ NTAs (Figure 4). This implies that more electron traps existed on the surface of pristine TiO_2_ than in TiO_2–*x*_ NTAs. The lower electron-trap density suggests that the charge recombination rates after NaBH_4_ treatment are lower. The RDB-PAS was found to be useful in understanding the change in the surface structure. RDB-PAS measurements of our samples were conducted to elucidate the change in the electronic states on the sample surfaces by analyzing the ERDT (energy-resolved distribution of electron traps (ETs)), i.e., vacant (electron unfilled) electronic states in TiO_2_ and TiO_2–*x*_ NTAs. As a principle of RDB-PAS, vacant electronic states or ETs are measured by filling them up; electron-filled ETs cannot be detected. Another interesting feature is that almost all of the detected ETs in ERDT measurements may be located on the surface, not the bulk.^34^ Therefore, this technique is sensitive to changes in the surface structure. As shown in Figure 4, the starting TiO_2_ showed two small peaks at ca. 3.3 and 4.0 eV, which are assignable to anatase and amorphous titania.^34^ The very low total density (TD) seems reasonable since the intensity of the TD depends strongly on the total amount of ETs (on the sample surface) in the NTA sample. After the NaBH_4_ treatment (TiO_2–*x*_), the 4.0 eV peak disappeared, and the 3.3 eV peak became almost negligible. There are two possible interpretations for this change: one is that the original ETs on TiO_2_ remained unchanged but were filled with electrons during the process so as not to be detected by RDB-PAS. Another interpretation is that NaBH_4_ treatment decomposed the ETs on TiO_2_. We also treated the TiO_2–*x*_ film with ozone to understand whether these created OVs are subject to recovery. The ERDT pattern of TiO_2–*x*_ after ozone treatment may suggest the change in ETs; two small peaks at 3.2 and 4.2 eV appeared, and a relatively large peak (though still very low density compared to powder samples of titania) was recorded at below 2.4 eV. One plausible explanation is that the original ETs on TiO_2_ were partly electron-filled by NaBH_4_ treatment without their structure and recovered by ozone treatment before RDB-PAS; a small shift of those peaks might be due to changes in their neighborhood. The other part of ETs that is not remaining might be decomposed. Considering the results of XPS studies showing increasing amounts of defects (OVs), those defects are not vacant electronic states and thereby not detected by RDB-PAS. It is suggested that the NaBH_4_ treatment increased the OVs, changed the surface structure, which decreased surface ETs, and changed the environments of the remaining ETs. In this study, the RDB-PAS patterns can be used as an indicator of the OVs in TiO_2_ and the activity of the sample in PEC-assisted catalysis.

**Figure 5:**
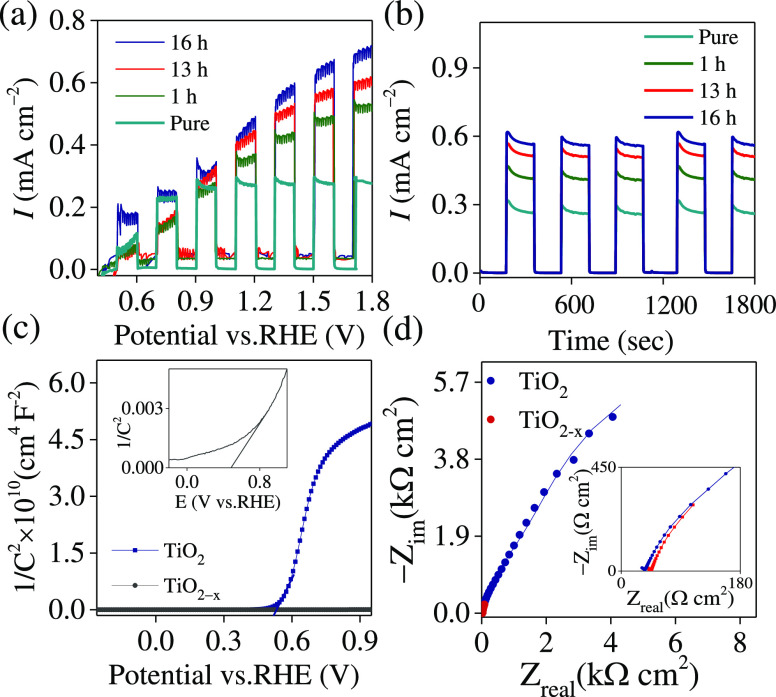
Comparison of TiO_2_ and TiO_2–*x*_ NTAs (reduced in 1 M NaBH_4_ for 16 h): (a) LSV in 0.1 M Na_2_SO_4_ (pH 5) recorded at 5 mV/s under chopped illumination (λ: 370 nm). Samples are treated in 1 M NaBH_4_ solution at different times. (b) Current vs time recorded at 1.5 V vs RHE. (c) M–S plots in 0.1 M Na_2_SO_4_ (the inset is a magnified area of TiO_2–*x*_ NTAs). (d) The Nyquist plot at 1.23 V vs RHE under illumination.

We have studied the photocurrents of TiO_2–*x*_ NTA photoanodes as a function of NaBH_4_ treatment time. All of the TiO_2–*x*_ NTAs have higher photoactivity than the pristine TiO_2_ NTAs, as shown in Figure 5a. The sample treated for 16 h yields a maximum photocurrent density for water oxidation which is 0.5 mA cm^–2^ at 1.23 V vs RHE. The inclusion of lower electron traps (e.g., oxygen traps) in TiO_2–*x*_ NTAs leads to enhanced charge separation, resulting in a higher photocurrent density. It is feasible to adjust the density of electron-trap states by producing oxygen vacancies on the catalyst’s surface.^40^ A longer NaBH_4_ treatment for 24 h causes a higher level of OVs in TiO_2–*x*_ (33.1%) compared to 16 h (31.6%), which gives lower PEC efficiency (Figures S2d and S3). Similar results for water oxidation are reported on surface fluorinated TiO_2_ nanoporous films.^41^ Corby et al.^42^ reported the performance of nanostructured WO_3_ films with different concentrations of bulk oxygen vacancies and found that the medium OV concentration gave the maximum photocurrent. High levels of defects result in a greater probability of trap-mediated recombination. The holes that are trapped deep in the material are unable to contribute to water oxidation, and thus higher incidences of trap-assisted recombination result in lower photocurrents.^41,42^

The chronoamperometry tests (*I*–*t*) were used to compare the photoresponses of TiO_2–*x*_ and TiO_2_ NTAs under chopped UV illumination at 1.5 V vs RHE (Figure 5b). Without illumination, the current values were nearly zero, while the photocurrent rapidly increased to a steady-state value upon illumination. This trend was reproducible for several on/off cycles, and the highest photocurrent value of 0.6 mA cm^–2^ at 1.5 V vs RHE was recorded with the TiO_2–*x*_ NTAs treated for 16 h. It is possible to estimate the flat-band potential (*V*_FB_) of the used semiconductor by measuring the capacitance of an electrode. According to the depletion layer model, the capacitance of the semiconductor space charge layer (*C*) depends on the applied potential (*V*) and can be estimated from the M–S equation^32,42^
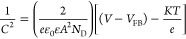
2where *e* is the electron charge (1.60 × 10^–19^C), ε_0_ is the vacuum permittivity (8.85 × 10^–12^ F·m^–1^), ε present the dielectric constant of TiO_2_ (ε = 10),^14^*A* is the surface area of the electrode, *k* is the Boltzmann constant (1.38 × 10^–23^ J K^–1^), *N*_D_ is the donor density, and *T* is the working temperature (298 K). For TiO_2_ and TiO_2–*x*_ NTAs, the V_FB_ was determined to be 0.54 and 0.44 V vs RHE, respectively, which are close to the literature-reported values^14^ (Figure 5c). The more negative *V*_FB_ of the TiO_2–*x*_ NTAs could be ascribed to the presence of Ti^3+^ due to improved carrier transport.^41,43−45^ It is worth mentioning that the *V*_FB_ determined from M–S measurements are different from those determined using XPS. In the case of the M–S study, the value is for the system where the electrode is in contact with the electrolyte and the solvent. The positive plot slopes of both TiO_2_ and TiO_2–*x*_ NTAs suggest that the material is an n-type semiconductor with electrons as the major charge carriers. The TiO_2_ NTA electrode showed a strong relationship between capacitance and applied voltage, indicating that the capacitance was controlled by the space charge layer.^15^ The slope of the M–S plots was also used to measure the donor density of the NTAs (Figure 5c). In particular, the reduced TiO_2–*x*_ NTAs have much smaller slopes, implying that donor concentrations have increased. The donor densities were estimated using the equation
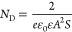
3

The calculated donor densities of the pristine and TiO_2–*x*_ NTAs are 4.95 × 10^19^ and 8.58 × 10^22^ cm^–3^, respectively. The increased *N*_D_ in TiO_2–*x*_ NTAs is associated with the increase in OVs that serve as electron donors.^46^ Furthermore, the increased donor density is projected to shift the Fermi level of TiO_2_ into the conduction band. On the other hand, the upward shift of the Fermi level facilitates the charge separation.^47^ A typical Nyquist plot of pristine and reduced TiO_2_ NTAs obtained at 1.23 V vs RHE in 0.1 M Na_2_SO_4_ solution is shown in Figure 5d.^30^ Clearly, the TiO_2–*x*_ photoanode presents a smaller arc, resulting in a decreased charge transfer resistance (*R*_ct_).^10^ Consequently, the reduced TiO_2–*x*_ NTA photoanode had a higher charge separation efficiency.

### 3.2 PEC Degradation of the B41 Dye

**Figure 6:**
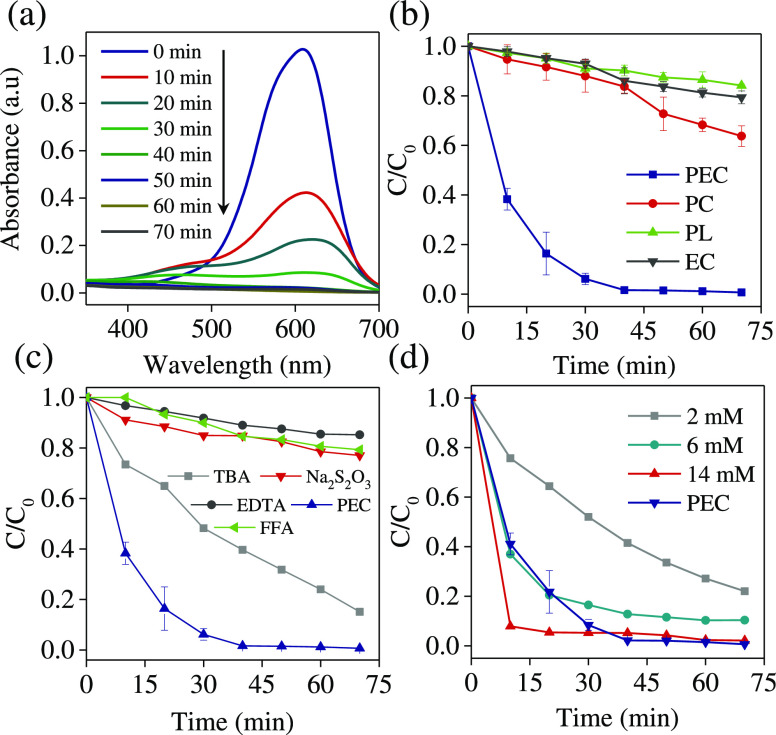
(a) Absorption spectra of the B41 dye recorded during PEC experiment. (b) Normalized concentration (*C*/*C*_0_) of the B41 dye vs time for (a) EC, PC, PL, and PEC processes and (c) PEC degradation in only 10 mM NaCl or in a mixture of 10 mM NaCl and a scavenger: 0.1 M TBA, 0.1 M EDTA, 0.1 M Na_2_S_2_O_3_, and 10 mM FFA, pH 5. (d) Comparison of B41 degradation using PEC and chemical oxidation at different NaOCl concentrations. All PEC experiments were conducted using the TiO_2–*x*_ thin film.

We used textile dye B41 as a model pollutant in PEC degradation studies. The absorption spectra of the B41 dye in 10 mM NaCl solution at various PEC degradation (at 1.5 V vs RHE) intervals using TiO_2–*x*_ are given in Figure 6a. The degradation kinetics of B41 was analyzed using pseudo-first-order kinetics: −ln(*C*/*C*_0_) = *kt*, where *C*_0_ is the initial concentration, *C* is the concentration at sampling time *t*, and *k* is a pseudo-first-order rate constant calculated from the slope of the straight lines for each treatment. The PEC degradation using the TiO_2–*x*_ film with Cl^–^ ions gave *k* = 0.0858 min^–1^, which was found to be higher than that for pure TiO_2_ (*k* = 0.0189 min^–1^) (Figures S4b and S5).

To compare the activities of treated and untreated TiO_2_ films, the quantum yield (Φ) during the PEC reaction was studied. The Φ is defined as the ratio between the number of B41 dye molecules being degraded to the number of photons absorbed by the photocatalyst^48^
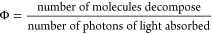
4Based on the literature, the photodegradation rate of dye molecules under the monochromatic light source can also be used for the calculation of quantum yield using the following equation
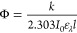
5Here *I*_0_ represents the incident light intensity at a specific wavelength (in this study, 370 nm, 20 W, ∼0.7 × 10^–6^ Einstein L^–1^ s^–1^), ε_λ_ is the molar absorptivity of B41 chosen at 610 nm (27 934 cm^–1^ M^–1^), and *l* is the path length (3 cm) of the reaction container (75 mL) being illuminated.^48−50^ The calculated Φ for the treated and untreated TiO_2_ are 0.635 and 0.139 mol Einstein^–1^, respectively. The Φ of the TiO_2–*x*_ film is 3 times higher than the pristine sample. Furthermore, the activity of the TiO_2–*x*_ film was also studied under electrocatalytic (EC), photocatalytic (PC), and photolysis (PL) conditions (Figure 6b). The results show that the PEC process allows complete decolorization (e.g., degradation) of the B41 dye in 70 min. The degradation efficiency (*D*) was calculated using the following equation: *D* (%) = (*C*_0_ – *C*)/*C*_0_ × 100, where *C*_0_ is the concentration of B41 at zero time (*t* = 0 min), and *C* is the concentration at the reaction time specified.^32^ While the EC treatment (at 1.5 V vs RHE) allows only 18.7% degradation efficiency of B41, the PC and PL approach yields 33.3 and 16.2% in 70 min, respectively. In PEC mode, the degradation of B41 is much more efficient and reaches 99.3%. The PEC degradation rate constant is much higher than the EC (*k* = 0.0033 min^–1^), PC (*k* = 0.0059 min^–1^), and PL (*k* = 0.0025 min^–1^) (Figure S5). The mineralization of the B41 dye was demonstrated using the COD method.^32^ The COD value in PEC-treated solution was initially 67 mg L^–1^, which dropped to 18 mg L^–1^ after 70 min. Thus, the PEC approach gives partial mineralization of the B41 dye.

To gain insight into the mechanism of PEC dye degradation using TiO_2_ (Figure S4c) and TiO_2–*x*_ NTAs (Figure 6c), different molecules/species were used as scavengers for hydroxyl (^•^OH) radicals (TBA), photogenerated holes (EDTA), formation of singlet oxygen (FFA), and RCS (Na_2_S_2_O_3_). It has been demonstrated that the oxidation of organics using the PEC process can proceed via direct or indirect mechanisms. Direct oxidation occurs with electron transfer between the electrode and the organic molecules. On the other hand, indirect oxidation involves strong oxidant species such as RCS and reactive oxygen species (ROS), which are generated in situ during the course of the PEC process.^32^ In the presence EDTA, the degradation of the B41 dye is slow, which indicates that the ROS and RCS generation are suppressed. Adding in the solution Na_2_S_2_O_3_ scavenger inhibits the generation of RCS, which in turn gives lower dye degradation efficiency. However, it is interesting to note that when TBA is added as a ^•^OH radical scavenger, the degradation of the B41 dye occurs at significant levels.^32^ Moreover, to check the formation of singlet oxygen (^1^O_2_), reactive species for the degradation of organics, FFA was used as a scavenger. Often, the generation of ^1^O_2_ in metal oxides is associated with OVs where the rate constants equal to 1.5 × 10^10^ M^–1^ s^–1^ were reported.^51^ Negligible variations in the B41 degradation were observed, indicating that ^1^O_2_ originated from the lattice oxygen atoms in the TiO_2–*x*_ NTA photoanode rather than from dissolved oxygen in the solution.^52^ The PEC degradation efficiency of B41 in the presence of 10 mM FFA (with 10 mM NaCl) using TiO_2–*x*_ was only 11% and is much slower compared to pristine TiO_2_ NTAs, which give 45%. The lower degradation efficiency with TiO_2–*x*_ suggests that ^1^O_2_ species are scavenged by FFA. To better confirm the generation of ^1^O_2_, XPS was used to investigate the surface chemical composition before and after the PEC degradation of B41. For O 1s spectra of TiO_2–*x*_, the relative content of lattice oxygen was decreased from 59 to 52% (Figure S6). This suggested that the OVs were involved in the PEC degradation of the B41 dye. We can conclude that during the PEC process with the TiO_2–*x*_ photoanode in 10 mM NaCl solution, RCS and ^1^O_2_ species form, and this causes the degradation of the B41 dye.

To assess the role of RCS, we mimic the PEC conditions by adding different amounts of NaOCl (2, 6, and 14 mM) to the pure B41 dye solution (Figure 6d). It is worth mentioning that HOCl is the dominant form of NaOCl at pH = 5. Approximately 6 mM NaOCl was found to be sufficient for the decolorization of the B41 dye (under dark), which is comparable to the PEC system. This indicates that the decolorization of B41 is mainly driven by in situ generated RCS (e.g., HOCl).

As reported previously on the PEC decomposition of azo dyes, applied anodic potential can significantly affect the efficiency of dye degradation.^53^ In this study, by increasing the bias potential, the PEC degradation of the B41 dye increases (Figure S7a). As a rule of thumb, higher anodic potentials increase the photocurrent (Figure S7b).^32,54^

**Figure 7:**
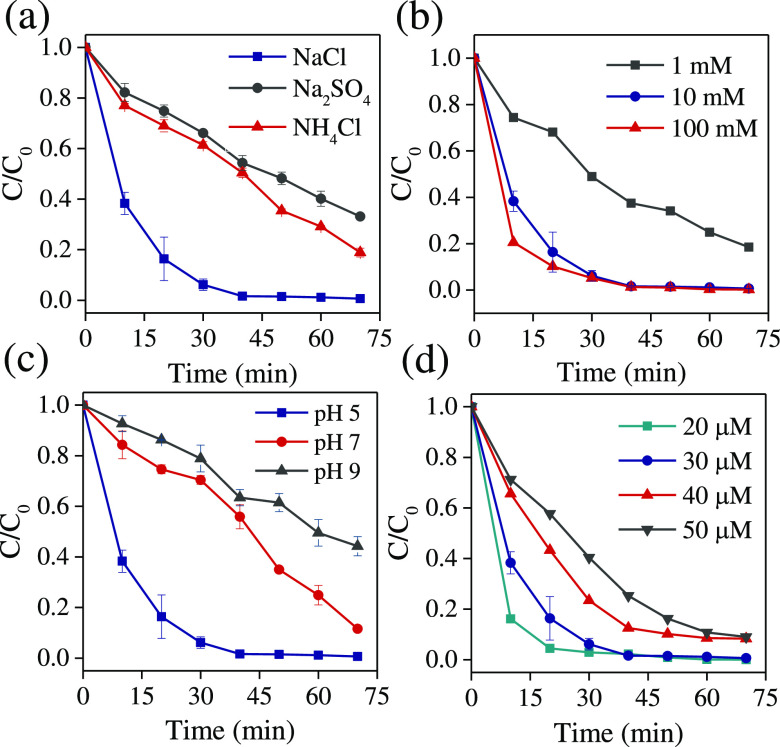
Normalized dye concentration vs time recorded using TiO_2–*x*_ NTAs (PEC) at applied 1.5 V vs RHE in (a) different electrolytes (10 mM), (b) the NaCl electrolyte (different concentrations), (c) different pH values, and (d) varying dye concentrations in 10 mM NaCl.

For comparison, the PEC degradation experiments were carried out in three different electrolytes: NaCl, NH_4_Cl, and Na_2_SO_4_ (Figure 7a). The LSV characteristics of TiO_2–*x*_ NTAs recorded in these electrolytes, together with the Tafel plot, are shown in Figure S8. As expected, the fastest degradation of the B41 dye occurs in the presence of the NaCl electrolyte. The degradation proceeded much more slowly in NH_4_Cl since the oxidation of Cl^–^ competes with the ammonium cation (NH_4_^+^). The oxidation of NH_4_^+^ ions can occur either using direct or indirect routes.^32^ We ascribed the slow dye degradation in the Na_2_SO_4_ electrolyte to the sluggish generation of ^•^OH and SO_4_^•–^ species. Figure 7b shows the effect of the NaCl concentration on the degradation efficiency of B41 with TiO_2–*x*_ NTA photoanode. With increasing the Cl^–^ concentration, a faster degradation is observed because of increased levels of RCS. Further, we also studied the effect of pH on B41 dye degradation at three different pH values (Figure 7c). The degradation process of the B41 dye was the fastest at pH 5. The explanation for this difference is that at low pH values (pH >3), Cl_2_ dissolves to yield the powerful HOCl oxidant, while at higher pH values, to less effective OCl^–^ ion. Another reason for the slower reaction at higher pH values (pH 9) is that the *V*_FB_ edge potential of TiO_2–*x*_ NTAs moves in the negative direction, reducing the driving force of Cl^–^ ion oxidation.^55^ Series of PEC experiments were also performed at different initial concentration ranges of the B41 dye, from 20 to 50 μM (Figure 7d). As the initial concentration of B41 increases, the degradation of the dye becomes slower, which is also observed during the degradation of other organics.^29^ The kinetics of the azo dye photooxidation can be described using the Langmuir–Hinshelwood (L–H) kinetic model^56^
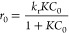
6
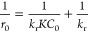
7where *r*_0_ is the initial rate of reaction (M min^–1^), *k*_r_ is the L–H reaction rate constant (M min^–1^), *K* is the L–H adsorption equilibrium constant (M^–1^), and *C*_0_ is the initial concentration of pollutant (M). The L–H kinetic model assumes surface chemical reactions between adsorbed species. The dye molecules adsorbed on the electrode surface can be directly oxidized with the holes (h^+^) from the semiconductor. However, parallel reactions with adsorbed Cl^–^ ions or ^•^OH are required for PEC dye degradation. The oxidation of Cl^–^ species by the PEC process can generate RCS, which can contribute to indirect dye degradation.^57,58^Figure S9 shows a plot of 1/*r*_0_ vs 1/*C*_0_ for the degradation of the B41 dye in the PEC system. From the intercept (1/*k*_r_), the apparent rate constant was calculated to be 1.0 × 10^–6^ M min^–1^. Based on the slope, the *K* value was determined to be 2.47 × 10^4^ M^–1^. The high value of the constant *K* assumes a strong interaction of B41 with the TiO_2–*x*_ surface. The constant *K* can also be related to the standard free energy of adsorption, Δ*G*^0^, using the following equation^59^
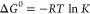
8where *T* represents the absolute temperature (298.15 K) and *R* is the gas constant (8.314 J mol^–1^ K^–1^). The calculated value of Δ*G*^0^ for this system is equal to −25.1 kJ mol^–1^. The negative value of *G*^0^ indicates that the adsorption of the B41 molecule on the TiO_2–*x*_ surface occurs spontaneously. This result is also consistent with the literature reports for the adsorption of charged molecules on solid surfaces where the *G*^0^ values are in the order of −20 kJ mol^–1^ or higher.^32,59^ On the other hand, there are limited experimental studies on the adsorption of Cl^–^ onto TiO_2_. Li et al. carried out density functional theory (DFT) calculations on the adsorption of Cl^–^ onto TiO_2_ with oxygen vacancies, where they reported a moderate Δ*G*^0^ of −0.51 eV (−49.2 kJ mol^–1^).^60^ After adsorption of Cl^–^ on TiO_2_, the Cl^–^ is oxidized by the holes of TiO_2_ spontaneously giving a Δ*G*^0^ value of −123 kJ mol^–1^. Therefore, in our system, the competitive adsorption of the B41 dye and Cl^–^ is expected to occur on the TiO_2–*x*_ surface.

LC-MS was used for the identification of degradation products during PEC treatment of the B41 dye (10 mM NaCl). The peak area of the molecular cation (M^+^) of the B41 dye at *m*/*z* = 371^+^, C_19_H_23_N_4_O_2_S, with retention time around 5.7 min is 218 counts at 0 min treatment (PEC treatment time), 51 counts in a sample at 10 min, and in less than 5 counts in a sample at 20 min. The disappearance of the absorption peak of the B41 dye at 60 min elution time indicates the breaking of the azo bond in the dye molecule (Figure S10). The chlorinated by-product with the protonated molecular ion (MH^+^) at *m*/*z* = 405, C_19_H_22_N_4_O_2_SCl, is given at a retention time of 3.7 min (Figure S11). This product is present only in samples at 20, 30, and 40 min. It is worth mentioning that sometimes in the presence of Cl^–^ ions during EC and PEC processes, the degradation of organic pollutants could form toxic chlorinated molecules or chlorates.^55,61^ However, the formation of chlorinated products is absent in the present system after 50 min of PEC treatment.

### 3.3 PEC Degradation of IBF

**Figure 8:**
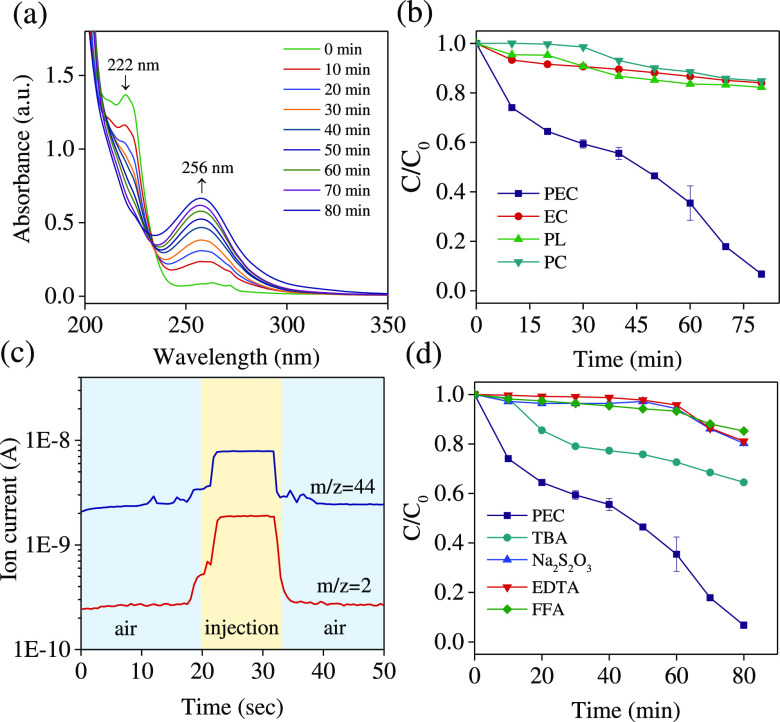
(a) Absorption spectra of IBF recorded during the PEC experiment. (b) Normalized concentration (*C*/*C*_0_) of IBF vs time for EC, PC, PL, and PEC. (c) Ionic current vs time of H_2_ and CO_2_ recorded using MS. (d) Normalized concentration of IBF during PEC degradation (at 1.5 V vs RHE) in the presence of 10 mM NaCl and different scavengers: 0.1 M TBA, 0.1 M EDTA, 0.1 M Na_2_S_2_O_3_, and 10 mM FFA, pH 3. All PEC experiments were conducted using TiO_2–*x*_ NTAs.

The progress of IBF degradation was monitored using UV–vis spectroscopy (Figure S12). The initial IBF solution exhibits an absorption band at 222 nm (Figure 8a). The intensity of this band decreases markedly during PEC oxidation due to oxidative ring opening. Degradation of IBF using different methods: EC, PC, PL, and PEC, is given in Figure 8b. Complete PEC degradation of 0.2 mM of IBF was observed after 80 min. On the other hand, within the same irradiation period, the PC and EC methods removed just 30 and 14% of the IBF, respectively. Moreover, the rate constant of the pseudo-first-order kinetics for IBF degradation using the PEC process was found to be 0.0377 min^–1^ and is higher than the pristine TiO_2_ NTAs (*k* = 0.0129 min^–1^) (Figures S13 and S14a). The PEC degradation of IBF using TiO_2–*x*_ in this work was faster than other photoanodes reported in the literature (Table S2). For example, using the Ti/Zn-TiO_2_ catalysts during IBF degradation under PEC condition yielded *k*_1_ = 0.0008 min^–1^, which is slower than the present study.^62^

In addition, the degradation quantum yield of IBF was also calculated using eq 4, and the results demonstrate an excellent photoelectrocatalytic performance of TiO_2–*x*_ (1.219 mol Einstein^–1^) compared to TiO_2_ (0.268 mol Einstein^–1^). Quantum yields of IBF degradation by the TiO_2–*x*_ were higher than those in the UV-based advanced oxidation processes (Table S3). The quantum yield of IBF degradation can be further increased by the presence of OVs to fully utilize the returned radiant energy of evanescent waves in TiO_2–*x*_ and to increase its photocatalytic reactive sites.^63^ The evolved gasses in the anodic chamber were used as an indicator to follow the oxidation of IBF organics using mass spectrometry (MS). The presence of H_2_ (*m*/*z* = 2) collected from the cathodic chamber and CO_2_ (*m*/*z* = 44) from the anodic chamber is given in the MS in Figure 8c. The formation of CO_2_ was ascribed to the mineralization of IBF during the PEC process.

It is interesting to note that the PEC degradation of organic pollutants could involve ROS. It is well known that TBA is a good scavenger of ^•^OH radicals (rate constant of 5 × 10^8^ M^–1^ s^–1^).^64^ Sun et al. demonstrated that ^•^OH was dominated for the degradation of IBF using the Cu_2_O/TiO_2_ photoanode.^22^ The PEC degradation of IBF in the presence of TBA was less efficient than in the system without adding a scavenger molecule (Figure 8d). This result indicates that the contribution of ^•^OH radicals during the degradation process is negligible with added TBA.^32^ Some researchers have reported that HOCl can dissociate to ^•^OH and Cl^•^ upon absorption of the UV photon in the region 200–400 nm.^61^ During the PEC degradation process, the following reactions may occur (eqs 9–11).
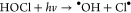
9
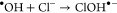
10
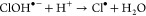
11

Furthermore, coumarin was also used as a qualitative probe for ^•^OH radicals which gives highly fluorescent 7-hydroxycoumarin (7-HC, peak at ∼455 nm).^32,55^ The lack of emission peak from 7-HC in Cl^–^ containing electrolytes suggests that ^•^OH radical formation is suppressed (Figure S15). Therefore, RCS generated in the PEC system plays a major role in the degradation of organic pollutants compared to the systems which generate only ^•^OH radicals. The degradation of IBF was also significantly inhibited with the addition of EDTA or Na_2_S_2_O_3_ scavengers. This implies that these molecules block the formation of RCS and ROS. Moreover, FFA blocked the degradation efficiency of IBF using TiO_2–x_ as a photoanode compared to TiO_2_ (Figure S14b). In the TiO_2–*x*_ system, the formation of ^1^O_2_ is suppressed, and that is why the degradation of IBF is slow. Therefore, we can conclude that using the TiO_2–*x*_ film in 10 mM NaCl, both RCS and ^1^O_2_ species evolve, and these participate in IBF degradation.

**Figure 9:**
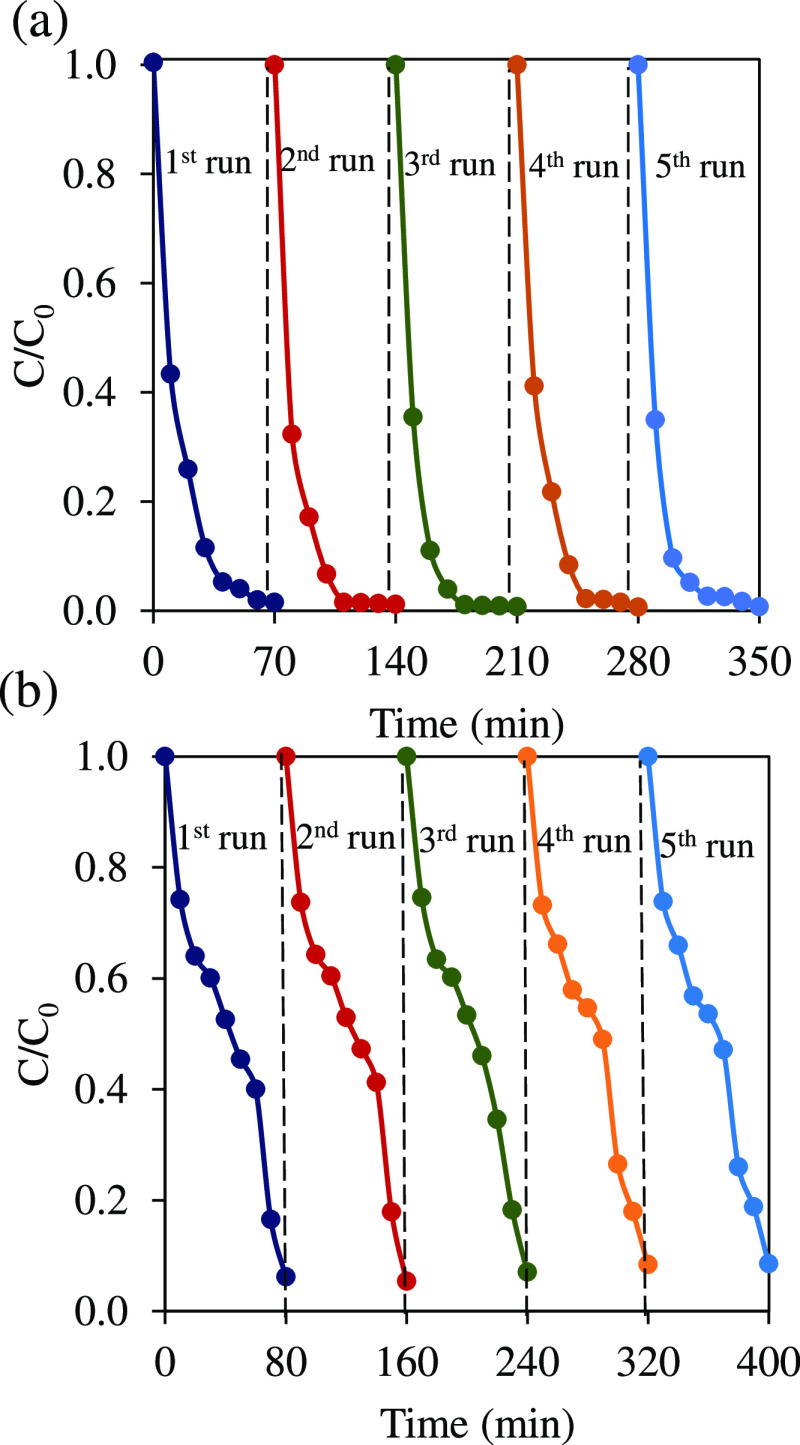
PEC degradation of (a) the B41 dye and (b) IBF for five runs at 1.5 V vs RHE in 10 mM NaCl.

Reusability tests using TiO_2–x_ NTAs for IBF and B41 dye degradation were repeated up to five cycles in the PEC system (Figure 9a,b). The TiO_2–*x*_ film showed over 90 and 99% degradation efficiency of IBF and the B41 dye, respectively. To strengthen this conclusion, RDS-PS measurement of TiO_2–*x*_ NTAs showed an insignificant change between the fresh and used catalysts, suggesting the good chemical stability of reduced TiO_2_ NTAs (Figure S16). These results show that the TiO_2–*x*_ film is stable against corrosion and could be reused in PEC studies.

**Figure 10:**
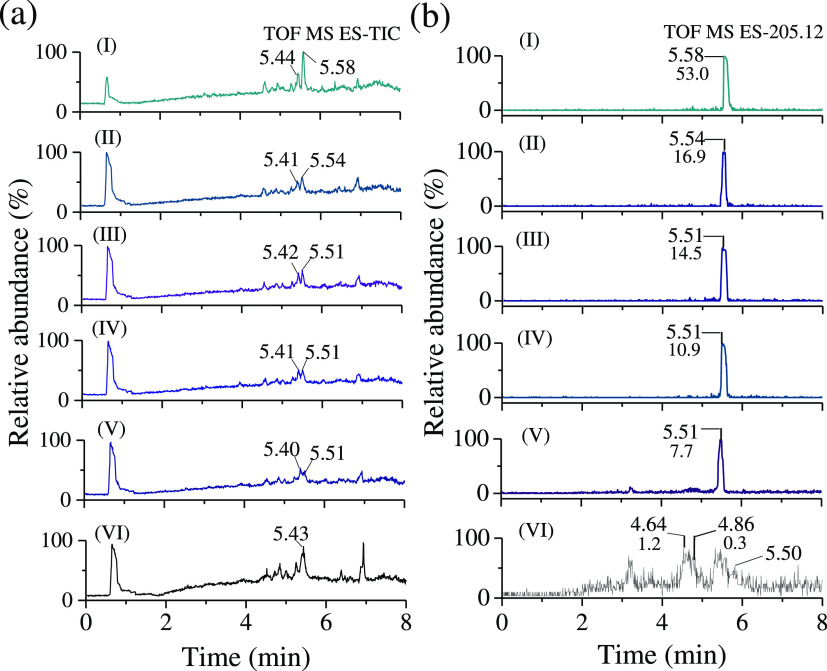
(a) Total negative ion chromatograms of IBF samples (I → VI, from top to bottom) from 0 to 75 min with a step of 15 min. (b) LC-MS ion chromatograms of IBF, MH^–^*m*/*z* = 205 at different PEC degradation times (0–75 min).

LC-MS was used to check the degradation levels and formation of any by-products during the PEC process. The LC-MS chromatogram taken from a reference IBF sample (0 min sample) shows a well-defined peak at 5.5 min elution time. The disappearance of the same IBF peak in the sample, which is PEC treated for 75 min, indicates that fragmentation of the IBF molecule occurs (Figure 10a,b). Figure S17 shows a plot of IBF concentrations at various reaction times. The concentration of IBF reduced from 45.6 mg L^–1^ (*t* = 0 min) to 0 mg L^–1^ during the 90 min treatment. For IBF degradation, the TiO_2–*x*_ photoanode provides good catalytic activity. Electrospray ionization mass spectroscopy (TOF-MS-ES) for species with a mass-to-charge (*m*/*z*) equal to 205 ([M – H]^−^) also decreases the intensity during the 70 min PEC treatment (Figure 10b).

### 3.4 Mechanism of PEC Degradation

**Figure 11:**
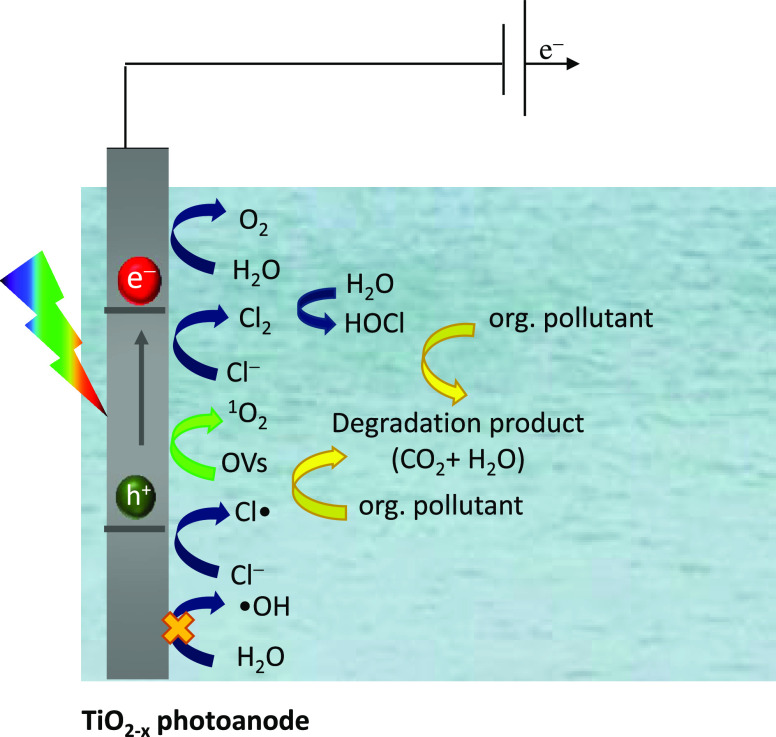
Working mechanism of PEC degradation of organic pollutants (B41 and IBF) using the NaCl electrolyte.

Based on the reported experimental results, we proposed a possible path for the PEC degradation of organic pollutants (Figure 11). The degradation process is primarily associated with the photoinduced charges (e^–^ and h^+^) in TiO_2–*x*_ catalysts.^1^ The photogenerated holes on the photoanode can oxidize chloride to Cl^•^ species which competes with H_2_O oxidation (eqs 12–14). The reaction of Cl^•^ with chloride ions could give free chlorine (eqs 15–17).^32,55^
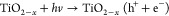
12
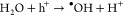
13
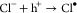
14
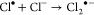
15
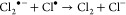
16
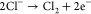
17
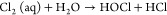
18

Moreover, as shown in eq 18, in situ generated Cl_2_ will hydrolyze to HOCl in an acid medium (pH 5). The latter molecule alone or in the presence of Cl^•^ and Cl_2_^•–^ species will participate in the oxidation of organic molecules. In parallel to indirect oxidation, there is also a second pathway for the generation of reactive species, which involves OVs. It has been demonstrated that OVs have the tendency to yield active oxygen (O*). Further, O* is transformed into ^1^O_2_ in the presence of NaCl.^52^ Negligible variations in the IBF and B41 degradation were observed, indicating that ^1^O_2_ originated from the lattice oxygens in the TiO_2–*x*_ photoanode rather than from dissolved oxygen in the solution. Based on the results of the above analyses, it was concluded that the OVs in TiO_2–*x*_ can enhance the production of ^1^O_2_ and RCS, therefore improving the PEC degradation efficiency of B41 and IBF.

### 3.5 Phytotoxicity Test Using *L. sativum* L.

Seed germination and root growth of *L. sativum* were carried out to study the phytotoxic effect of tested B41 dye and IBF solutions (Figures S18 and S19). Inhibition (%) values are 39.2 and 15.4% for the untreated and PEC-treated B41 solutions, respectively. Furthermore, for IBF, the inhibition value for the treated sample obviously reduced (from 36.0 to 4.8%). The descent in the inhibition value shows that the toxicity of B41 and IBF decreased under the experimental conditions used using the PEC system.

**Figure 12:**
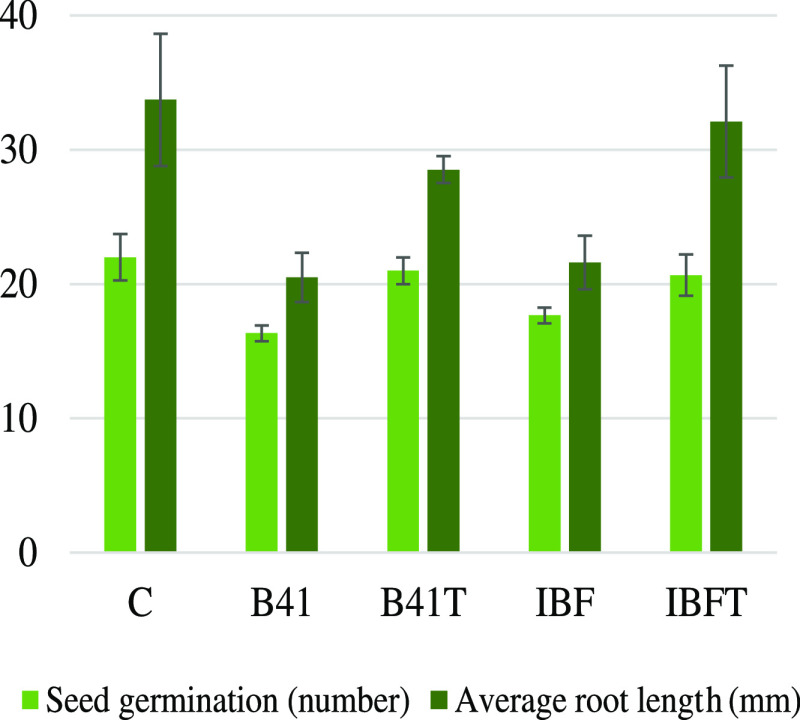
Seed germination test for toxicity analysis of B41 and IBF solutions.

Table S4 shows the germination index (GI) values of the untreated and PEC-treated B41 and IBF samples. Zucconi et al., have mentioned that the GI values are used to classify phytotoxicity.^65^ When the GI is close to 80%, an analyte is considered nontoxic, and the GI value closer to 100% indicates a plant-stimulating effect (Figure 12). In our case, the GI determined after PEC treatment of both pollutants are above 80%, which means that at the studied concentration ([B41] = 0.03 mM, [IBF] = 0.2 mM), there is an absence of phytotoxicity.^66^ For comparison, Table S4 shows the relative germination percentage (RGP) and relative radicle growth (RRG) values from which the GI value was determined. The results display that the treated simulated water gives lower values for all parameters.

## 4 Conclusions

In this study, TiO_2–*x*_ NTAs were prepared by using a one-step anodization method and a chemical etching method using NaBH_4_. It was found that NaBH_4_ treatment increases the electron donor density in TiO_2_ NTAs by creating surface OVs. The introduced surface OVs account for the negative-shifted flat-band potential, decreased surface recombination centers, and decreased electron-trap state density. The TiO_2–*x*_ NTAs exhibit excellent PEC performance. As a comparison with the PC and EC processes, the PEC treatment allows faster degradation of IBF (90%) and B41 dye (99%) efficiency. As shown by LC-MS, the TiO_2–*x*_ NTA photoanode yields efficient and complete degradation of organic pollutants. In the presence of the Cl^–^ electrolyte, COD measurement confirms the mineralization efficiency of the B41 dye after the PEC process. Phytotoxicity test using *L. sativum* L. shows a descend in the inhibition value, which proves that the toxicity of the B41 dye and IBF were decreased after PEC treatment. The toxicity test using *L. sativum* showed higher values for the relative germination percentage, relative radicle growth, and germination index, which are parameters describing efficient plant growth.

## Supporting Information Available

RDB-PAS, XPS, LC-MS, phytotoxicity test conditions; PEC cell used in the B41 and IBF degradation studies; XPS spectra; additional (photo)electrochemical data; PEC degradation data; LC-MS data of B41 and IBF degradation; and fluorescence emission spectra of coumarin (Figures S1–S19); Structural formula of the B41 dye and IBF; overview of the PEC degradation of IBF by various photocatalysts; quantum yields of Ibuprofen degradation by different processes; and value percentage of toxicity tests (Tables S1–S4) (PDF)
