# Supplementary material for: Defective TiO2 Nanotube Arrays for Efficient Photoelectrochemical Degradation of Organic Pollutants
Source: ACS Omega. 2023 Jun 7;8(24):21605–17. doi: 10.1021/acsomega.3c00820 (PMC10286085; doi:10.1021/acsomega.3c00820)
Supplement: Supplementary file 1 — ao3c00820_si_001.pdf [file ao3c00820_si_001.pdf]

Supporting information

Defective TiO_2 Nanotube Arrays for Efficient Photoelectrochemical Degradation of Organic Pollutants

Manel Machreki,[1] Takwa Chouki,[1] Georgi Tyuliev,[2] Dušan Žigon,[3] Bunsho Ohtani,[4] Alexandre Loukanov,[5] Plamen Stefanov,[6] and Saim Emin[1,*]

[1] Materials Research Laboratory, University of Nova Gorica, Vipavska 11c, 5270, Ajdovščina, Slovenia

[2] Institute of Catalysis, Bulgarian Academy of Sciences, Acad. G. Bonchev St., Bldg. 11, Sofia 1113, Bulgaria

[3] Institute "Jožef Stefan", Jamova 39, 1000, Ljubljana, Slovenia

[4] Catalysis Research Center, Hokkaido University, N21, W10, 001-0021 Sapporo, Japan

[5] Department of Chemistry and Materials Science, National Institute of Technology, Gunma College, 580 Toriba, Maebashi, 371-8530 Gunma, Japan

[6] Institute of General and Inorganic Chemistry, Bulgarian Academy of Sciences, Sofia, 1113, Bulgaria

*Corresponding author. E-mail address: saim.emin@ung.si

Experimental section

Material characterizations

The reversed double-beam-photoacoustic spectroscopy (RDB-PAS) measurements were performed ausing the TiO_2 and TiO_{2-x} NTAs samples were placed in a PAS cell (PASCL02-009). The RDB-PAS was equipped with a MEMS (micro-eletro-mechanical system) microphone (SparkFun MEMS Microphone Breakout, INMP401) and a quartz window on the upper side [1]. For RDB-PAS measurements, the PAS cell was filled with methanol-saturated nitrogen, and two light beams were introduced simultaneously using a UV quartz combiner light guide (Moritex MWS5-1000S-UV3). One was a 625-nm light beam from an LED modulated by a digital function generator (NF Corporation DF1906) at 35 Hz, and the other was continuous monochromatic light from a monochromator (Bunkokeiki M10) equipped with a 500-W mercury-xenon lame (Hamamatsu Photonics L8288) with a wavelength scanning from 600 nm to 250 nm with 5-nm steps. The PA signal was detected by a digital lock-in amplifier (NF Corporation LI5630). The raw spectrum obtained was differentiated from the lower-energy side and calibrated with the reported total electron-trap density in units of $\mu mol\ g^{-1}$ measured by a photochemical method to obtain an energy-resolved distribution of electron traps (ERDT) pattern [1].

The XPS measurements were performed in the analysis chamber of the electron spectrometer ESCALAB-Mk II (VG Scientific) with a base pressure of ~ 5 x 10^{-8} Pa. The samples in "as prepared" form were mounted on a conductive adhesive tape and C1s, O1s, Ti2p photoelectron and TiLMM Auger spectra were recorded by using AlK_{α} radiation. All spectra were calibrated by using C 1s peak at 285.0 eV as a reference. The surface composition was evaluated from the photoelectron intensities divided by the corresponding photoionization cross sections taken from Scofield [2].

LC-MS analysis

Liquid chromatography coupled with mass spectrometry (LC-MS) measurements were performed with a Water Acquity binary pump based ultra-performance liquid chromatography (UPLC) system (Waters, Milford, USA) binary pump with an Ascentis express C-18 column (2µm, 100 x 2,1 mm i.d). LC column with mobile phases consisting of 0.1 % formic acid in water (A) and acetonitrile (B). The elution gradient was produced by a linear increase from 95% A to 95% B in 7 min and then back to initial conditions at 8 min. The flow rate of mobile phase was 0.3 mL min^{-1}. Injected volume of 1 to 5 µl samples are introduced through electrospray ionization source (ESI). Compressed nitrogen (99.999%, Messer Slovenia) was used as both the drying and the nebulising gas. The nebulizer gas flow rate was set to 20L h^{-1} and the de-solvation gas flow rate to 600L h^{-1}. A cone voltage of 20V and a capillary voltage of 2.5kV in negative ion mode (ESI-) for IBF and 3kV in positive ionization mode (ESI+) for IBF were used. The de-solvation temperature was set to 3000°C and the source temperature to 1200°C. Mass spectra were acquired in centroid mode over an m/z range of 50-1000 in scan time 0.2s and inter scan time 0.02s. For identification and characterization of organic compounds a mass resolution of 10000 was applied for accurate high-resolution mass measurements.

Phytotoxicity test using *Lepidium sativum L*

Lepidium sativum L. was used to assess the acute toxicity of B41 and IBF before and after treatment. B41 and IBF model solutions were prepared and degraded as described in PEC section. In the experiment 15 *L. sativum* seeds were evenly placed on a filter paper in Petri dish (Ø90 mm) and exposed by adding 3 ml of treated and untreated B41 model solution (sample B41T and B41 before, respectively)), and treated and untreated IBF model solution (sample IBFT and IBF, respectively). Control (C) was also included by adding distilled water to the seeds. Incubation was performed at room temperature and after 72h seed germination and root growth were evaluated. The experiment was done in triplicate for each treatment. Relative

germination percentage (RGP), relative radicle growth (RRG) and germination index (GI) were calculated according to the methodology described [3].

Inhibition (%) was calculated as follow:

$$Inhibition\ (\%) = \frac{Root\ lenght\ (C) - Root\ length\ (sample)}{Root\ length\ (C)} \times 100$$

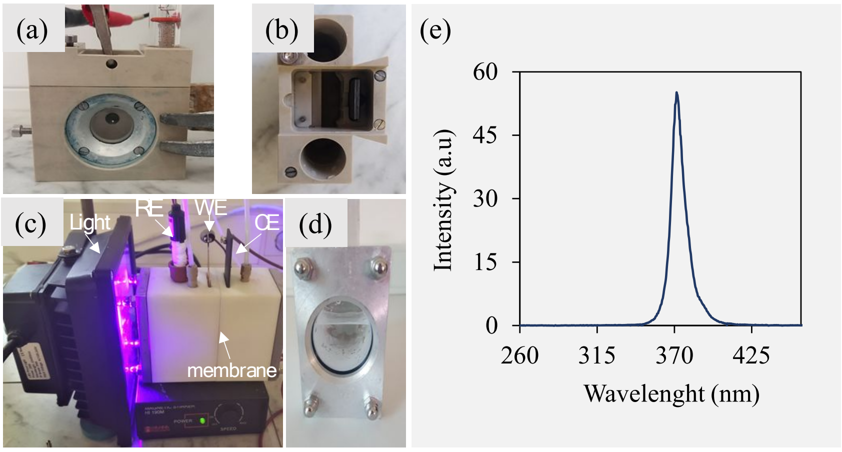

Figure S1. Photograph of the cappuccino cell used to record the EIS and LSV measurements in (a) side-view and (b) top-view. PEC cell used in the dye degradation studies: (c) complete view and (d) side-view of the quartz window (e) LED lamp spectrum used as a light source.

Energy-dispersive X-ray spectroscopy (EDS)

Energy-dispersive X-ray spectroscopy (EDS) analysis was used as a rough estimate to determine the atomic ratios of O and Ti elements. In the pristine TiO_2 TNAs the atomic ratio is 1.6 and is higher than in the reduced TiO_2 TNAs which yielded a vlue of 1.3 (Figure S2e, f). In our opinion the measured atomic ratio does not reflect the true value since the Ti signal from the foil may also contribute to the total Ti signal. Since both samples have similar thicknesses and morphology, we can say that there is decrease in O signal in the reduced TiO_2 NTAs sample which could be an indicator for the formation of oxygen vacancies.

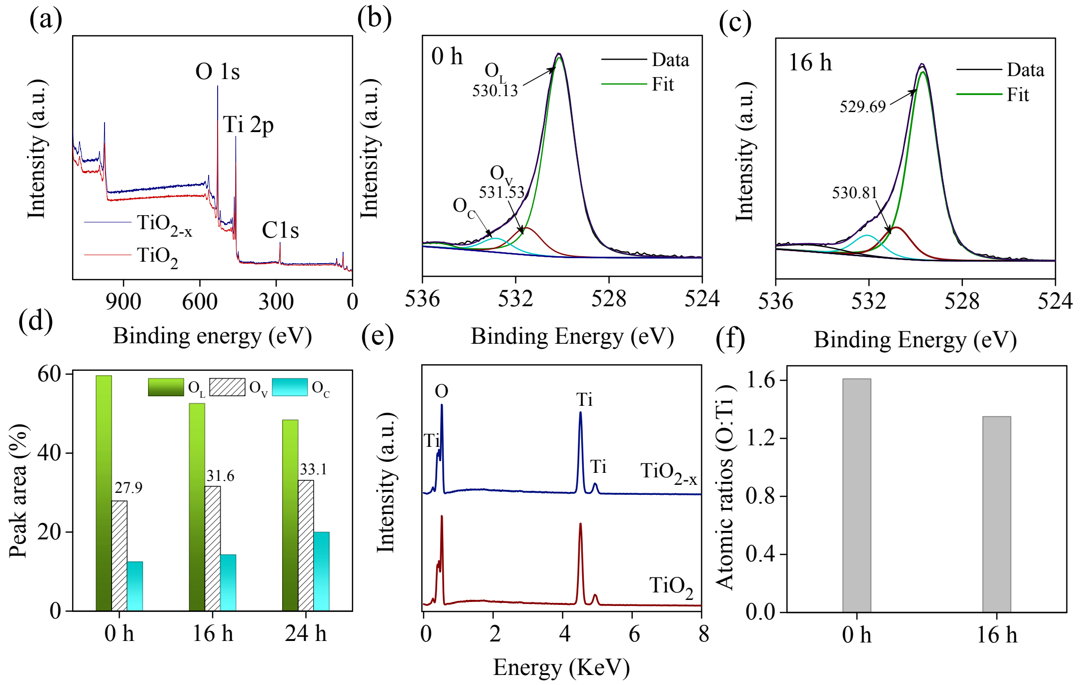

Figure S2. (a) XPS spectra of pristine TiO_2 and $NaBH_4$ treated TiO_{2-x} treated sample for 16h. (b-c) deconvoluted O 1s XPS spectra of TiO_2 NTAs at different treatment times where each spectrum is fitted with three components: chemisorbed oxygen (O_C), lattice oxygen (O_L), and oxygen vacancies (O_V). (d) The percentage of areas under the deconvoluted peaks for the three samples. (e) EDS spectra of the pristine TiO_2 and $NaBH_4$ treated TiO_{2-x} treated sample for 16h. (f) The corresponding variations of atom ratios of O:Ti elements.

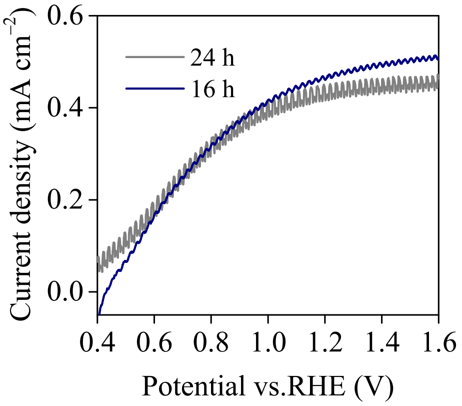

Figure S3. LSV of pristine TiO_2 and $NaBH_4$ treated TiO_{2-x} treated sample for 16 and 24 h in 0.1M Na_2SO_4 (pH 5) recorded at 5 mV/s.

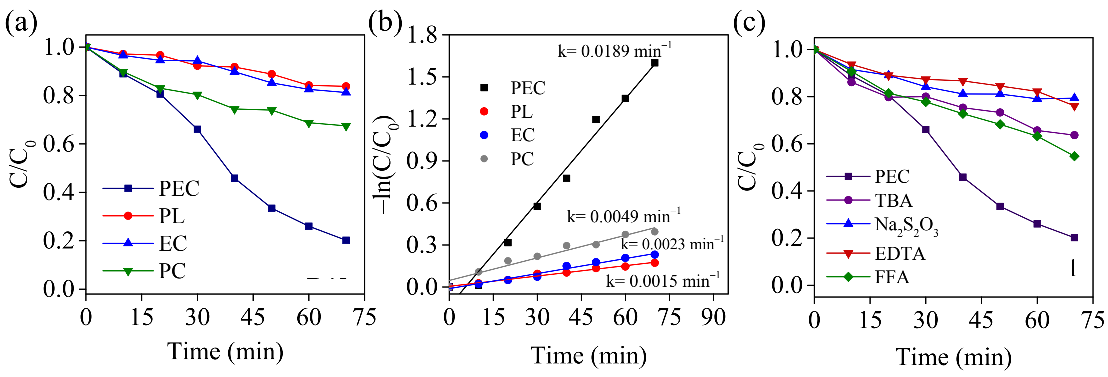

Figure S4. Normalized concentration (C/C_0) of B41 dye vs. time for (a) EC, PC, PL, and PEC. (b) Degradation rate constants of different conditions using TiO_2 in 10 mM NaCl. (c) PEC degradation using only 10 mM NaCl or a mixture of 10 mM NaCl and different scavengers: 0.1 M TBA, 0.1 M EDTA, 0.1 M $Na_2S_2O_3$, and 10 mM FFA. All experiments were conducted using TiO_2 thin film at pH 5.

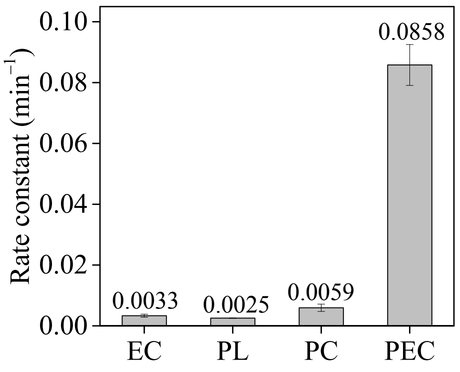

Figure S5. Average reaction rate constant (min^{-1}) for degradation of B41 dye using TiO_{2-x}.

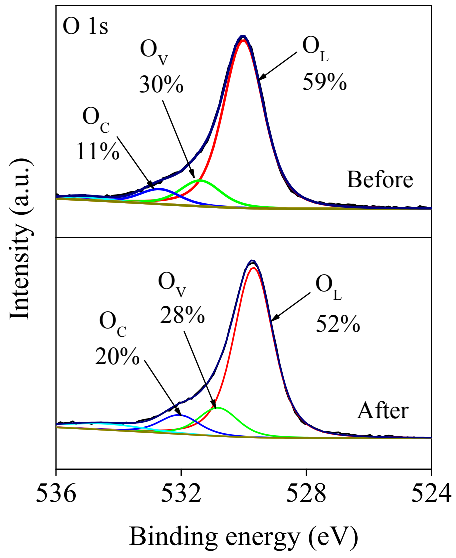

Figure S6. XPS spectra of TiO_{2-x} before and after degradation time.

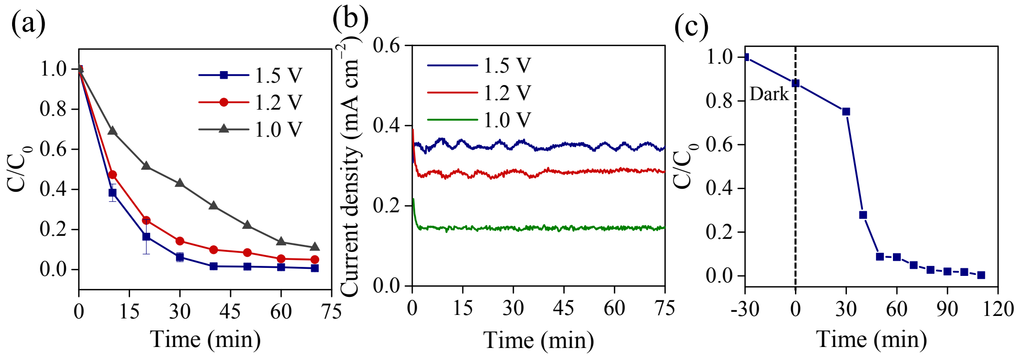

Figure S7. (a) Normalized Effect of applied potential using TiO_{2-x} NTAs. Current density vs. time recorded during degradation of B41 dye at different: (b) applied potential in 10 mM NaCl and (c) Normalized concentration vs time showing the contribution of B41 dye adsorption on the TiO_{2-x} NTAs under dark (30 min) and PEC degradation (0 to 110 min) using 10 mM NaCl and bias potential of 1.5 V vs. RHE.

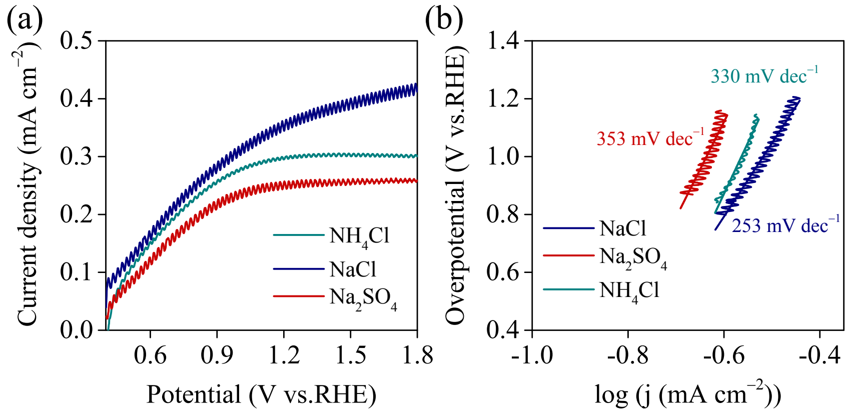

Figure S8. (a) LSV of TiO_{2-x} NTS photoanode (geometrical area 6 cm^2) in 0.01 M NaCl, Na_2SO_4 and NH_4Cl, respectively recorded at 5 mV s^{-1}. (b) Tafel plot of TiO_{2-x} NTS in different electrolytes as measured in (a).

Figure S8a shows LSV characteristics of TiO_{2-x} film in different electrolytes. The reactions can occur in a solution containing chloride ions:

$$2H_2O \rightarrow O_2 + 4H^+ + 4e^- \quad (1)$$

$$2Cl^- \rightarrow Cl_2 + 2e^- \quad (2)$$

The oxygen evolution reaction (OER) reaction (eq 1) is inherently slow because it is four-electron transfer technique, at the same time as the chlorine evolution reaction (CER) (eq 2) includes two electrons and is a far faster reaction. The Cl_2 generated dissolves to HOCl or OCl^- depending on the pH of solution. The higher current in NaCl electrolyte is attributed to both reactions: OER and CER. The pathway and kinetics of OER are typically investigated using semi-logarithmically plotted current-potential curve (eq 3), typically called a Tafel plot (Figure S8b).

$$\eta = a + b \cdot \log(j) \quad (3)$$

where j is the current density, a is the intercept and b is a coefficient, Tafel slope. Within the case of OER, the values of b are smaller than 500 mV dec^{-1} and gives facts approximately the mechanism of O_2 evolution. The values of b for efficient water oxidation catalysts are inside the range of 30 – 120 mV dec^{-1} [4]. When the values of b are high then these can be ascribed to various factors like (i) the observed reaction is restricted via mass transfer, (ii) surface diffusion or some chemical steps is the rate determining step, (iii) the accumulation of oxygen bubbles or reactive oxygen species which block the active surface of the electrode, etc [5]. The values of b in this work are 257 mV dec^{-1} (NaCl), 330 mV dec^{-1} (NH_4Cl), and 353 mV dec^{-1} (Na_2SO_4) and are much more efficient than the Tafel slopes reported in the literature [6]. However, these experimental values are lower than the data reported in the work of Zhu et al [4]. Worth mentioning that in NaCl and NH_4Cl electrolytes the Tafel slope are not entirely from the OER reaction but involves competitive oxidation reaction of Cl^- ions (CER). For efficient catalysts like RuO_2 the Tafel slopes for CER are usually under 100 mV dec^{-1} [7]. Herein, the Tafel slopes of TiO_{2-x} photoanode reported in Cl^- electrolyte is ascribed both to OER and CER. Since the Tafel slopes in Cl^- electrolyte is much lower it can be assumed that CER proceeds much efficiently than OER in the present system.

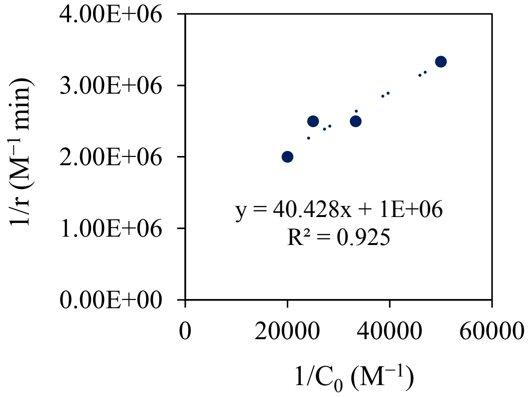

Figure S9. Determination of kinetic parameters for PEC degradation of B41 using Langmuir–Hinshelwood reciprocal rate dependence $1/r_0$ vs. $1/C_0$.

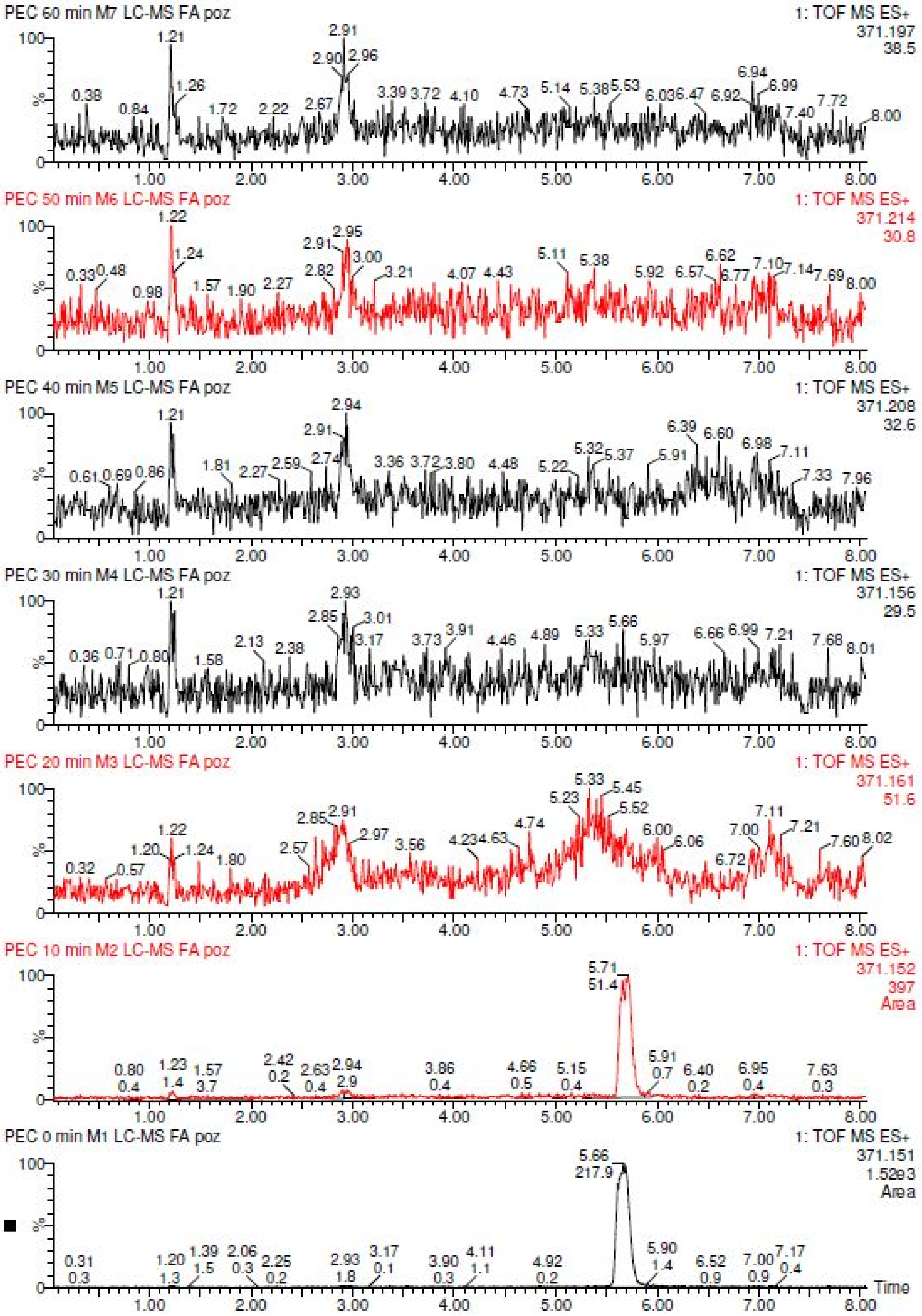

Figure S10. LC-MS ion chromatograms of B41 dye, Ion m/z=371^{+} chromatograms of samples at different PEC degradation time.

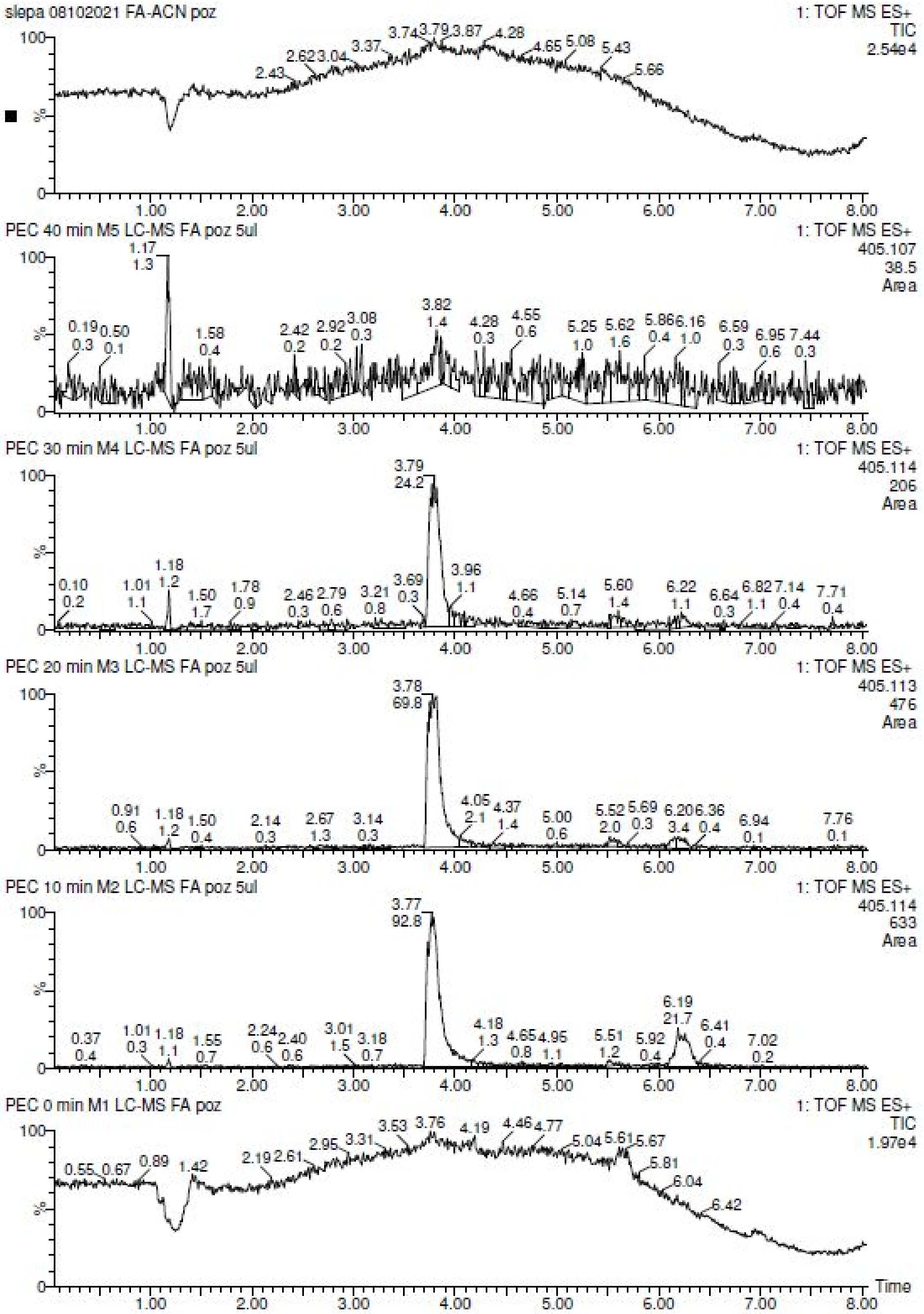

Figure S11. LC-MS ion chromatograms of chlorinated product, MH^+ at m/z=405 at retention time 3,7 min during degradation of B41 dye.

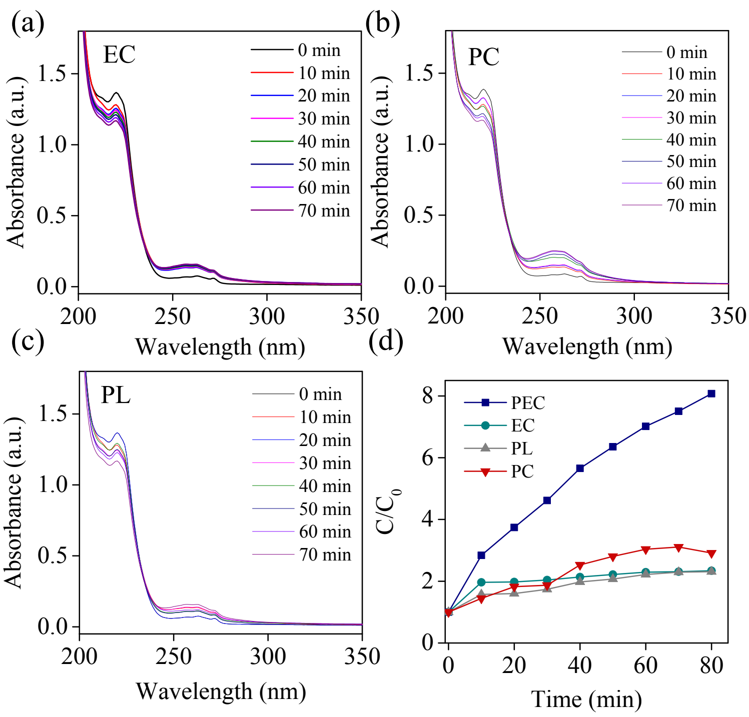

Figure S12. Absorption spectra of IBF recorded during (a) EC, (b) PC, and (c) PL experiment using TiO_{2-x}. (d) Normalized concentration (C/C_0) of formation of temporary products of IBP vs. time during EC, PC, PL, and PEC at absorbance λ_{max} 256 nm using TiO_2 NTAs.

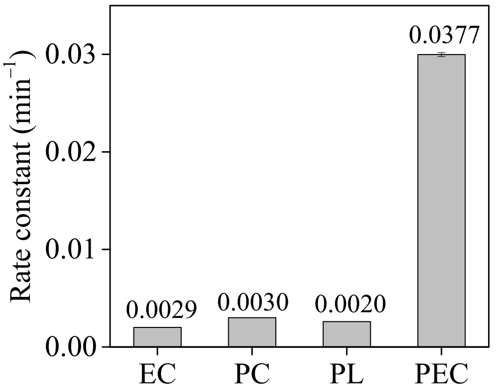

Figure S13. Average reaction rate constant (min^{-1}) for degradation of IBF using TiO_{2-x} in 10 mM NaCl.

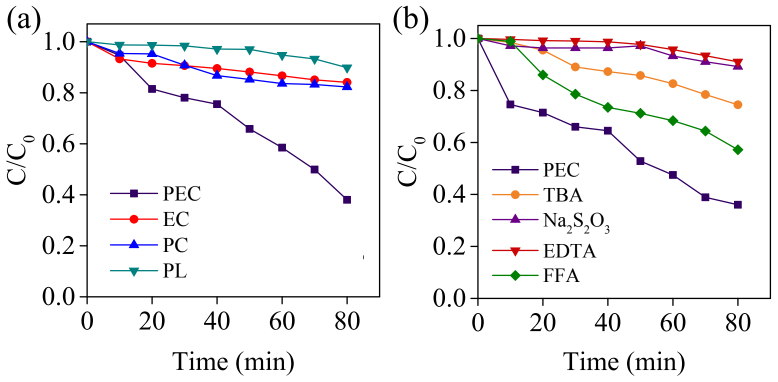

Figure S14. Normalized concentration (C/C_0) of IBF vs. time plotted after performing (a) EC, PC, PL, and PEC studies and (b) PEC experiment in 10 mM NaCl and different scavengers: 0.1 M TBA, 0.1 M EDTA, 0.1 M $Na_2S_2O_3$, and 10 mM FFA, pH 3. The experiments in (a) are conducted in 10 mM NaCl solution and using TiO_2.

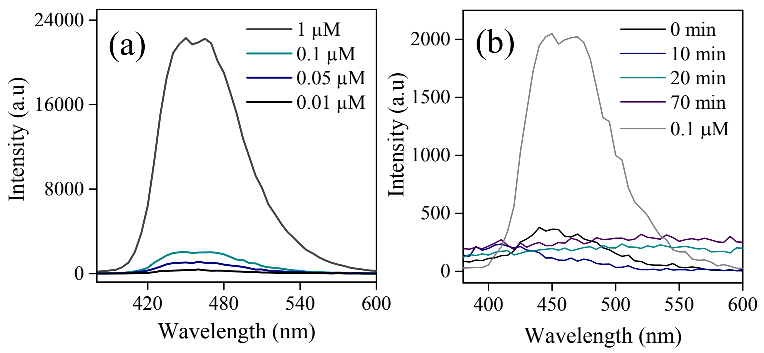

Figure S15. (a) The fluorescence emission spectra obtained (a) at different concentration of 7-HC and (b) during PEC treatment at different times with 30 µM coumarin as a probe molecule in 0.01 M NaCl. For comparison purpose in (b) is given also the intensity of 0.1 µM 7-HC standard.

It is well-known that •OH radicals react with coumarin molecules to form highly fluorescent 7-hydroxycoumarin (7-HC). The PEC experiment was carried out without B41 dye using 30 µM coumarin in 0.01M NaCl. The concentration of 7-HC was recorded via a fluorescence spectrophotometer at an excitation wavelength of 332 nm. Calibration plot at different concentrations of 7-HC is presented in Figure S15a. The lack of emission peak from PEC treated solutions confirm the absence of 7-HC (Figure S15b).

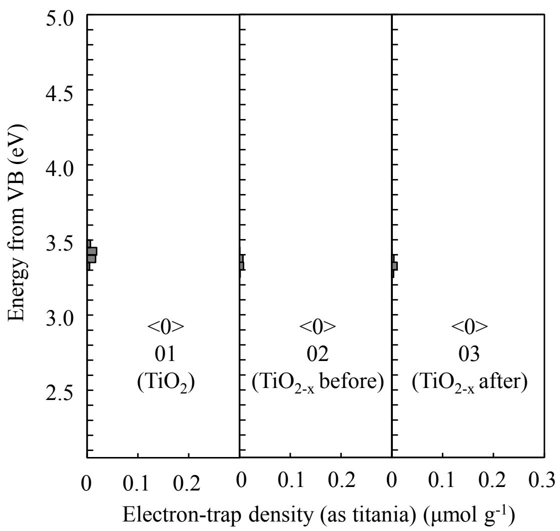

Figure S16. Representative ERDT patterns of TiO_2 and TiO_{2-x} NTAs before and after degradation. Figures in < > denote the total density of electron trap density in units of μmol g^{-1}.

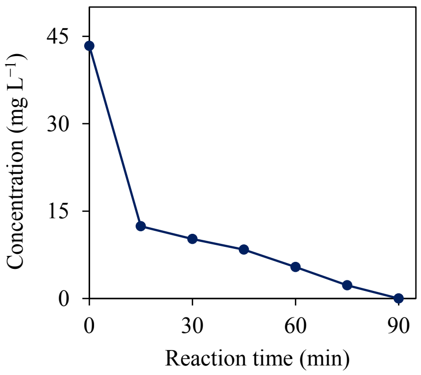

Figure S17. Decrease of IBF concentration during PEC degradation as determined using LC-MS.

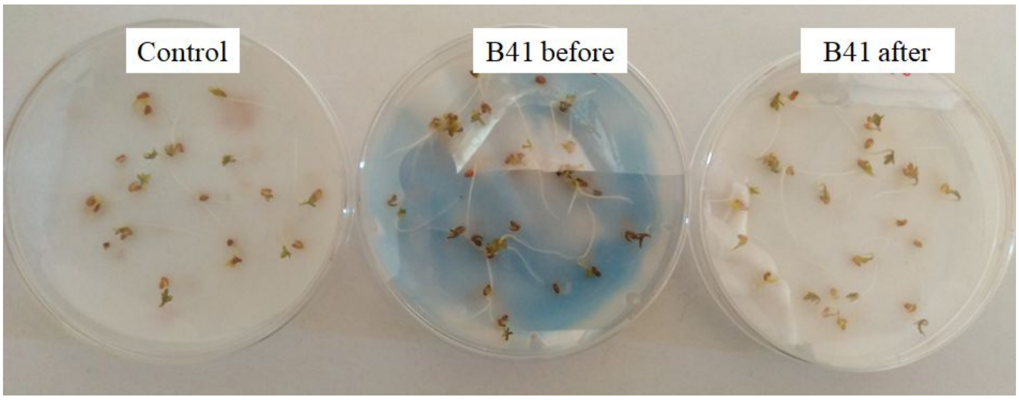

Figure S18. Images of *Lepidium sativum* L incubated in Petri dishes with B41 before and after PEC treatment.

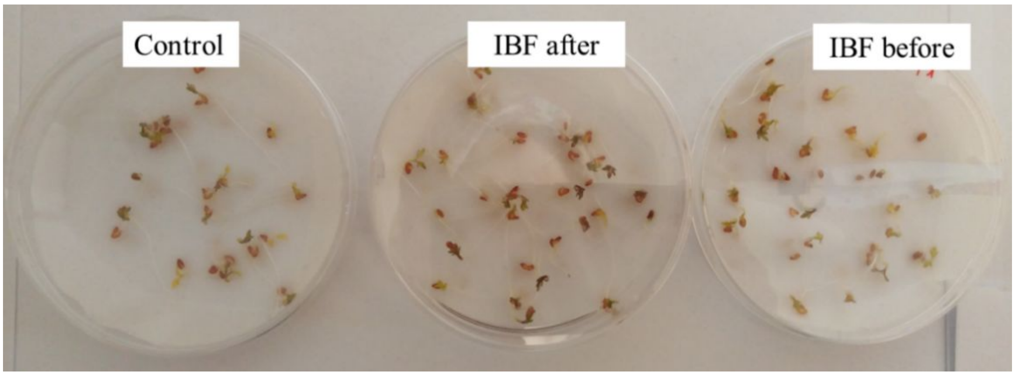

Figure S19. Images of *Lepidium sativum* L incubated in Petri dishes with IBF before and after PEC treatment.

Table S1. Characteristics of the B41 dye and IBF.

Parameters	Organic pollutants	
Commercial name	Bezacryl Blue GRL 300	Sodium ibuprofen
Chemical name	Basic blue 41 (B41)	Sodium ibuprofen (IBF)
Molecular weight (g/mol)	482.57	228.26
Molecular formula	$C_{20}H_{26}N_4O_6S_2$	$C_{13}H_{17}NaO_2$
Chemical structure	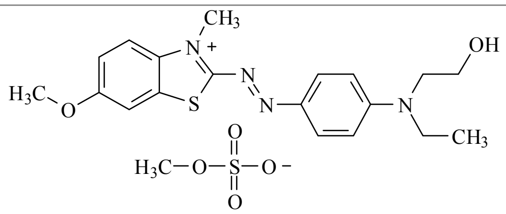	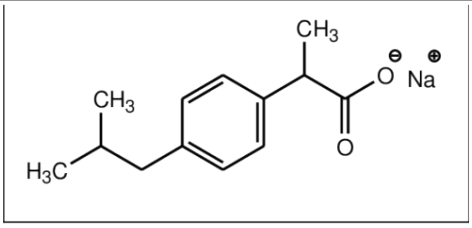

Table S2. Comparison of PEC degradation of IBF.

Catalysts	System	Concentration of pollutants	electrolyte	Light Source	Rate constant (min^{-1})	Degradation efficiency (time)	Ref.
**Ti/Zn-TiO$_2$**	PEC	50 mg L^{-1} IBF	0.035M Na_2SO_4	365 nm	0.0008	35% (180 min)	[8]
**Cu_2O/TiO$_2$**	PEC	10 mg L^{-1} IBF	0.1M NaCl	100 W Hg lamp	0.040	99% (120 min)	[9]
**BiOI/TiO$_2$**	PEC	5 mg L^{-1} IBF	0.1M NaCl	100W Hg light	0.0300	81% (120 min)	[10]

**Cu_2O/TiO_2**	PEC	10 mg L^{-1} IBF	0.1M NaCl	100W Hg light	0.0380	99% (90 min)	[11]
**Bi_2MoO_6–BDD**	PEC	10 mg L^{-1} IBF	0.1M Na_2SO_4	420 nm	–	82% (120 min)	[12]
**TiO_{2-x}**	**PEC**	**45.6 mg L^{-1} IBF**	**10mM NaCl**	**370 nm**	**0.0377**	**90% (80 min)**	**This work**

Table S3. Quantum yields of Ibuprofen degradation by different processes.

Processes	Quantum yield (mol $Einstein^{-1}$)	Irradiance (Einstein L^{-1} s^{-1})	$[IBF]_0$ (mg L^{-1})	Reference
Photolysis	0.33	1.2×10^{-6}	4.1	[13]
UV/H_2O_2	0.01	6.1×10^{-6}	2.1	[14]
Photolysis	0.17	2.1×10^{-6}	21	[15]
Photolysis	0.10	6.1×10^{-6}	2	[16]
UV/bromine	0.80	1.6×10^{-6}	41	[17]
UV/H_2O_2	0.20	4.5×10^{-5}	1.0	[18]
Photo-Fenton	0.25	4.1×10^{-7}	41	[19]
**PEC**	**1.22**	**0.7×10^{-6}**	**45.6**	**This work**

Table S4. Percentage of values before and after PEC treatment.

Samples	Inhibition	GI	RRG	RGP
B41	39.2	45.1	60.7	74.2
B41T	15.4	80.7	84.5	95.4
IBF	36.0	51.43	51.4	80.3
IBFT	4.8	89.4	89.4	93.9

References

[1] Nitta, A.; Takashima, M.; Murakami, N.; Takase, M.; Ohtani, B. Reversed Double-Beam Photoacoustic Spectroscopy of Metal-Oxide Powders for Estimation of Their Energy-Resolved Distribution of Electron Traps and Electronic-Band Structure. *Electrochim. Acta* **2018**, *264*, 83–90. https://doi.org/10.1016/j.electacta.2017.12.160.

[2] Scofield, J. H. Hartree-Slater Subshell Photoionization Cross-Sections at 1254 and 1487 EV. *J. Electron Spectros. Relat. Phenomena* **1976**, *8* (2), 129–137. https://doi.org/10.1016/0368-2048(76)80015-1.

[3] Mañas, P.; De las Heras, J. Phytotoxicity Test Applied to Sewage Sludge Using Lactuca Sativa L. and Lepidium Sativum L. Seeds. *Int. J. Environ. Sci. Technol.* **2018**, *15* (2), 273–280. https://doi.org/10.1007/s13762-017-1386-z.

[4] Zhu, K.; Ren, X.; Sun, X.; Zhu, L.; Sun, Z. Effect of Supporting Electrolyte on the Surface Corrosion and Anodic Oxidation Performance of Graphite Electrode. **2019**, 549–559.

[5] Katsuki, N.; Takahashi, E.; Toyoda, M.; Kurosu, T.; Iida, M.; Wakita, S.; Nishiki, Y.; Shimamune, T. Water Electrolysis Using Diamond Thin-Film Electrodes. J. Electrochem. Soc.

1998, 145 2358. https://doi.org/10.1149/1.1838643
[6] Comninellis, C.; Kapałka, A. Determination of the Tafel Slope for Oxygen Evolution on Boron-Doped Diamond Electrodes. **2008**, *10*, 607–610. https://doi.org/10.1016/j.elecom.2008.02.003.
[7] Exner, K. S.; Anton, J.; Jacob, T.; Over, H. Full Kinetics from First Principles of the Chlorine Evolution Reaction over a RuO_2 (110) Model Electrode. **2016**, *2* (110), 7501–7504. https://doi.org/10.1002/anie.201511804.
[8] Gomes, A.; Frade, T.; Lobato, K.; Jorge, M. E. M.; Da Silva Pereira, M. I.; Ciriaco, L.; Lopes, A. Annealed Ti/Zn-TiO_2 Nanocomposites Tested as Photoanodes for the Degradation of Ibuprofen. *J. Solid State Electrochem.* **2012**, *16* (6), 2061–2069. https://doi.org/10.1007/s10008-011-1608-0.
[9] Sun, Q.; Peng, Y. P.; Chen, H.; Chang, K. L.; Qiu, Y. N.; Lai, S. W. Photoelectrochemical Oxidation of Ibuprofen via Cu_2O-Doped TiO_2 Nanotube Arrays. *J. Hazard. Mater.* **2016**, *319* (1727), 121–129. https://doi.org/10.1016/j.jhazmat.2016.02.078.
[10] Chen, H.; Peng, Y. P.; Chen, T. Y.; Chen, K. F.; Chang, K. L.; Dang, Z.; Lu, G. N.; He, H. Enhanced Photoelectrochemical Degradation of Ibuprofen and Generation of Hydrogen via BiOI-Deposited TiO_2 Nanotube Arrays. *Sci. Total Environ.* **2018**, *633* (181), 1198–1205. https://doi.org/10.1016/j.scitotenv.2018.03.268.
[11] Chang, K. L.; Sun, Q.; Peng, Y. P.; Lai, S. W.; Sung, M.; Huang, C. Y.; Kuo, H. W.; Sun, J.; Lin, Y. C. Cu_2O Loaded Titanate Nanotube Arrays for Simultaneously Photoelectrochemical Ibuprofen Oxidation and Hydrogen Generation. *Chemosphere* **2016**, *150*, 605–614. https://doi.org/10.1016/j.chemosphere.2016.02.016.
[12] Zhao, X.; Qu, J.; Liu, H.; Qiang, Z.; Liu, R.; Hu, C. Photoelectrochemical Degradation of Anti-Inflammatory Pharmaceuticals at Bi_2MoO_6-Boron-Doped Diamond Hybrid Electrode under Visible Light Irradiation. *Appl. Catal. B Environ.* **2009**, *91* (1–2), 539–545. https://doi.org/10.1016/j.apcatb.2009.06.025.
[13] Vione, D.; Reddy, P.; Laurentiis, E. De; Minella, M.; Pazzi, M.; Maurino, V.; Minero, C.; Kouras, S.; Richard, C.; Giuria, V. P.; Analitica, C. Modelling the Photochemical Fate of Ibuprofen in Surface Waters. *Water Res.* **2011**, *45* (20), 6725–6736. https://doi.org/10.1016/j.watres.2011.10.014.
[14] Rongkui Su, Liyuan Chai, Chongjian Tang, B. L. and Z. Y. Comparison of the Degradation of Molecular and Ionic Ibuprofen in a UV/H_2O_2 System. *water Sci. Technol.* **2018**, 1–10. https://doi.org/10.2166/wst.2018.129.
[15] Szabó, R. K.; Megyeri, C.; Illés, E.; Gajda-schrantz, K.; Mazellier, P.; Dombi, A. Phototransformation of Ibuprofen and Ketoprofen in Aqueous Solutions. *Chemosphere* **2011**, *84*, 1658–1663. https://doi.org/10.1016/j.chemosphere.2011.05.012.
[16] Luo, S.; Wei, Z.; Spinney, R.; Zhang, Z.; Dionysiou, D. D.; Gao, L.; Chai, L.; Wang, D.; Xiao, R. UV Direct Photolysis of Sulfamethoxazole and Ibuprofen: An Experimental and Modelling Study. *J. Hazard. Mater.* **2018**, *343*, 132–139. https://doi.org/10.1016/j.jhazmat.2017.09.019.
[17] Guo, K.; Zheng, S.; Zhang, X.; Zhao, L.; Ji, S.; Chen, C.; Wu, Z.; Wang, D.; Fang, J. Roles of Bromine Radicals and Hydroxyl Radicals in the Degradation of Micropollutants by the UV/Bromine Process. *Environ. Sci. Technol.* **2020**, 54, 10, 6415–6426. https://doi.org/10.1021/acs.est.0c00723.
[18] Yuan, F.; Hu, C.; Hu, X.; Qu, J.; Yang, M. Degradation of Selected Pharmaceuticals in Aqueous Solution with UV and UV/H_2O_2. *Water Res.* **2009**, *43* (6), 1766–1774. https://doi.org/10.1016/j.watres.2009.01.008.
[19] Loaiza-ambuludi, S.; Panizza, M.; Oturan, N.; Oturan, M. A. Removal of the Anti-Inflammatory Drug Ibuprofen from Water Using Homogeneous Photocatalysis. *Catal. Today* **2014**, *224*, 29–33. https://doi.org/10.1016/j.cattod.2013.12.018.
